# Performance of binary prediction models in high-correlation low-dimensional settings: a comparison of methods

**DOI:** 10.1186/s41512-021-00115-5

**Published:** 2022-01-11

**Authors:** Artuur M. Leeuwenberg, Maarten van Smeden, Johannes A. Langendijk, Arjen van der Schaaf, Murielle E. Mauer, Karel G. M. Moons, Johannes B. Reitsma, Ewoud Schuit

**Affiliations:** 1 Julius Center for Health Sciences and Primary Care, University Medical Center Utrecht, Utrecht University, Utrecht, The Netherlands; 2 Department of Radiation Oncology, University Medical Center Groningen, Groningen University, Groningen, The Netherlands; 3 European Organisation for Research and Treatment of Cancer Headquarters, Brussels, Belgium

**Keywords:** Multi-collinearity, Prediction models, Normal-tissue complication probability models

## Abstract

**Background:**

Clinical prediction models are developed widely across medical disciplines. When predictors in such models are highly collinear, unexpected or spurious predictor-outcome associations may occur, thereby potentially reducing face-validity of the prediction model. Collinearity can be dealt with by exclusion of collinear predictors, but when there is no a priori motivation (besides collinearity) to include or exclude specific predictors, such an approach is arbitrary and possibly inappropriate.

**Methods:**

We compare different methods to address collinearity, including shrinkage, dimensionality reduction, and constrained optimization. The effectiveness of these methods is illustrated via simulations.

**Results:**

In the conducted simulations, no effect of collinearity was observed on predictive outcomes (AUC, *R*^2^, Intercept, Slope) across methods. However, a negative effect of collinearity on the stability of predictor selection was found, affecting all compared methods, but in particular methods that perform strong predictor selection (e.g., Lasso). Methods for which the included set of predictors remained most stable under increased collinearity were Ridge, PCLR, LAELR, and Dropout.

**Conclusions:**

Based on the results, we would recommend refraining from data-driven predictor selection approaches in the presence of high collinearity, because of the increased instability of predictor selection, even in relatively high events-per-variable settings. The selection of certain predictors over others may disproportionally give the impression that included predictors have a stronger association with the outcome than excluded predictors.

**Supplementary Information:**

The online version contains supplementary material available at 10.1186/s41512-021-00115-5.

## Background

Multi-collinearity between predictors is a common phenomenon in clinical prediction modeling, for example, in prediction of Alzheimer’s disease from MRI images [1], prediction of metabolic acidosis in laboring women that had a high-risk singleton pregnancy in cephalic presentation beyond 36 weeks of gestation [2], prediction of lung function in children [3], and prediction of complications of radiotherapy in cancer patients [4, 5]. Multi-collinearity is caused by dependence between predictors

[6]. When collinearity among predictors is high, the data in itself provides limited information on how the explained variance in the outcome should be distributed over the collinear predictor coefficients. In other words, there is not just one model, but there are multiple ways to assign coefficients that can predict the outcome in the data used to develop the model (almost) equally well [7]. Consequently, model coefficients of collinear variables generally show large variance (large standard errors) even in large data sets. Although this is generally not considered problematic with regard to predictive performance [8], it can result in unexpected coefficients for individual predictors, reducing the face-validity of the model in general, thereby potentially lowering the trust of clinicians in the model and their willingness to apply it in clinical practice [9, 10].

Two common methods to address collinearity are *predictor selection*, and *predictor averaging*. Both make strong assumptions about the predictive value of the collinear predictors. Predictor selection assumes that the excluded predictors have no added predictive value over the predictors that are retained in the model with respect to the outcome (essentially imposing coefficients of zero). Predictor averaging assumes that the averaged predictors have the same predictive relation to the outcome (imposing exact equivalence of the coefficients). In some cases, it is possible to convincingly motivate such assumptions using prior clinical knowledge or by resorting to data- driven approaches (e.g., backward selection). However, finding evidence in the data for such strong assumptions can be difficult, especially when collinearity is high, and the outcome is only weakly associated with the difference between collinear predictors. Therefore, further research into more sophisticated methods to address collinearity is needed.

This article is organized as follows: firstly, we describe different methods for handling multi-collinearity. Secondly, we compare the described methods via simulations in a case study on the development of models for the prediction of complications of radiotherapy in cancer patients, in terms of predictive performance, and in terms of coefficient estimation, including the choice of predictors in the final model. Lastly, we discuss and summarize our conclusions.

## Methods

### Compared prediction methods

#### Penalization of large coefficients

We assume the interest is in a binary outcome (*y*) and candidate predictors *X*. The aim is to estimate the risk of *y* conditioned on the predictor values, *P*(*y* = 1|***X***). As a base model, we assume standard logistic regression (LR), estimated by maximizing the likelihood of the outcome in the data used for model development. Mathematical details of all compared methods are present in Additional file 3.

In addition to the maximum likelihood of the outcome in the development data, approaches like **Lasso** and Ridge include the size of the model’s coefficients (excluding the Intercept) as an extra penalty for coefficient estimation. Adding this penalty results in models with smaller coefficients that make less extreme predictions (closer to the outcome proportion). The penalty can also reduce the variance in the estimated coefficients induced by collinearity. Although Lasso and Ridge have similar structure penalizing high regression coefficients, Ridge was originally designed to address collinearity, and Lasso to perform predictor selection in high-dimensional data. Lasso penalizes large coefficients linearly, by extending the cost function with the ℓ1-norm of the coefficients, which generally results in predictor selection of the most predictive features [11]. Ridge penalizes coefficient size quadratically, resulting in a grouping effect of collinear predictors, instead of selection [12]. In practice, the desire to perform predictor selection may be independent of the degree of collinearity present in the data, and rather to enhance usability of a more parsimonious prediction model. To facilitate a balance between predictor selection and grouping, the Elastic Net method was developed [13], which combines the penalties of Lasso and Ridge.

Penalization of coefficient size is a popular method in clinical prediction, aimed to improve predictive performance over maximum likelihood. Recent simulation studies suggest these penalization approaches often improve the predictive performance on average, but can show poor performance in small and low-dimensional datasets [14].

#### Dropout regularization

Dropout regularization is a method aimed directly at reducing co-adaptation of coefficients during model estimation, and is widely used for regularization of neural networks [15]. Co-adaptation refers to the degree to which the value of one regression coefficient depends on that of other coefficients. Dropout works in iterative gradient-based training procedures, like the one used in the current work (described in Additional file 3). When using Dropout, at each (gradient-based) learning step, all predictors have a non-zero probability δ to be dropped from the model, effectively selecting a random sub-model at each iteration. This selected sub-model is used to make predictions as part of that learning step, and the involved coefficients are updated accordingly. The coefficients selected at each step are updated independently of the dropped-out predictors, preventing co-adaptation in the final model. An alternative view to Dropout is to consider it as an efficient approximation to taking the mean over the predictions of an exponentially large set of sub-models, without having to estimate all those models individually.

Alternatively, Dropout can also be expressed as a penalty, which for logistic regression models is most similar to Ridge regularization, and includes a quadratic penalty on the size of coefficients. In contrast to Ridge, Dropout does not assign the penalty uniformly across the predictors. Dropout rewards infrequent predictors that enable the model to make confident predictions (predicted risks close to 0 or 1) whenever the predictor of interest is active [16].

#### Dimensionality reduction

The multi-collinearity of predictors may be due to shared dependence on a smaller set of unobserved underlying variables, that could themselves be related to the outcome. Principal component analysis (PCA) can reduce the dimensionality of the original predictor space, to obtain a smaller set of variables that explain (most of) the variance in the original predictors, but is in itself uncorrelated. These uncorrelated variables, the principal components, can be used as input to a logistic regression model to relate them to the outcome. This combination of PCA with logistic regression is called (incomplete) principal component logistic regression (PCLR) [17–19]. With regard to the original model, the effect of using PCLR is that predictors that correlate strongly, and are thus likely related the same principal components, obtain similar coefficients.

In this study, we focus on linear PCA as this gives the possibility to rewrite the PCLR model to an equivalent logistic model from the original predictors to the outcome (details on this are given in Additional file 3). This enables direct comparison of the coefficients with the other methods, and reduces the importance of interpretability of the principal components, as we can always observe the coefficients of each of the predictors in the final model.

Linear autoencoders (LAE) are similar to PCA but do not find the exact same projection as PCA. However, their components span the same directions [20]. In contrast to PCA or LAE, which determine the components based on the explained variance in the original predictors irrespective of the outcome, we extend the training criterion of LAE to find components that not only explain the variance of the original predictors but are also predictive of the outcome (from now on referred to as *linear autoencoder logistic regression*; LAELR). The relative importance of (1) explaining the variance in the predictors, and (2) maximizing the likelihood of the outcome, is determined by an additional parameter that (like the number of used components) needs to be tuned. How to tune such parameters is discussed later in the article. To summarize, LAELR can be seen as a compromise between PCLR and logistic regression (a more detailed formulation can be found in Additional file 3).

#### Constrained optimization

Besides penalizing the absolute size of coefficients, as in Lasso or Ridge, other penalties or criteria can be incorporated, possibly using knowledge from the clinical domain or setting. For example, in some cases, it may be valid to assume a priori that it is unlikely that certain predictors have a negative predictive relation with the outcome (e.g., in the later described case study one could assume that increasing radiation dosage to healthy tissue does not reduce the risk of complications). Encouraging the non-negativity (**NN**) of certain coefficients can be modeled by adding a penalty for negative coefficient values to the maximum likelihood criterion [21]. Alternatively, if the non-negativity constraints are to be respected at all times they can be incorporated as hard constraints during the maximum likelihood estimation of the model through, for example, gradient projection [22].

If the additional assumptions based on domain knowledge are correct and complementary to the information already present in the training data, incorporating them can reduce the coefficients’ search space. This may prevent selection of implausible models that satisfy maximum likelihood but are in fact inconsistent with clinical knowledge, and consequently reduce the coefficient variance due to multi-collinearity.

### Motivating example

#### Clinical background

Cancer patients receiving radiation therapy often experience complications after the therapy due to radiation damage to healthy tissue surrounding the tumor. For example, common complications for head and neck cancer patients are xerostomia (decreased salivary flow resulting in dry mouth syndrome), or dysphagia (swallowing problems). Prediction models, called normal-tissue complication probability (NTCP) models, are used to predict the risk for individual patients of developing complications after radiation-based therapy, based on patient, disease, and treatment characteristics including the dose distributions given to the healthy tissue surrounding the tumor, the so-called organs at risk (OAR) [23–25]. Besides informing patients about the expected risks of radiation-induced complications, NTCP models are clinically used to guide treatment decisions by looking at the difference in predicted risk of complications (∆NTCP) between treatment plans: sometimes by pair-wise treatment plan comparison [26–28], but also for complete treatment plan optimization [29, 30], where the planned dosage is adjusted to minimize the risk of complications, by minimizing the model-predicted NTCP, while maintaining tumor control.

For this setting, proper handling of collinearity is crucial, as in the process of treatment plan optimization unexpected coefficients may result in steering dosage to OAR that due to collinearity may not seem important (e.g., if the estimated coefficients are zero or negative), but in fact are associated with increased complication risks.

#### Simulation study

We planned and report the simulation study using the ADEMP (Aims, Data-generating mechanisms, Estimands, Methods, and Performance measures) strategy, following Morris and colleagues [31].

#### Aims

The aims of this simulation study are to

i. Study the *effect of collinearity* on development of clinical prediction models in terms of discrimination, calibration, and coefficient estimation in low dimensional settings (the number of predictors is smaller than the number of events).
ii. Compare the *effectiveness of eight methods* in handling the potentially negative effects of collinearity (logistic regression, Lasso, Ridge, ElasticNet, PCLR, LAELR, Dropout, and non-negativity-based constrained optimization).

#### Data-generating mechanisms

The simulations are based on four prediction modeling settings: mimicking two outcomes in our motivating example (xerostomia and dysphagia), and two predictor sets per outcome: a smaller predictor set with less collinearity, where the given radiation is only indicated by the mean dose per OAR, and a larger predictor set with higher collinearity, where more detailed dose-volume predictors are added as well^1^. These four initial settings are in Table 1: *A* and *C* for the settings with small predictor sets, and *B*_△_ and *D*_△_ for the larger predictor sets. For these four settings, predictor data were simulated from a m ulti-variate normal distribution, using the means and covariance matrix of the observed predictors of 740 head-and-neck patients (with primary tumor locations: pharynx, larynx, or the oral cavity) that underwent radiotherapy at the University Medical Center Groningen (UMCG), and were selected for having no missing data in the predictors or outcome. In simulations, to establish a ground-truth relation between predictors and outcome one often sees that all logistic regression coefficients are fixed to a certain constant (say, a log odds ratio of 0.2 ). Here, to establish realistic regression coefficients of the data generating model, the simulated ground-truth relation between predictors and outcome is constructed by fitting a logistic regression with Ridge penalization on the corresponding real data from our motivating example^2^. These regression coefficients are then used to generate our simulation study data to which the compared methods are fit, and are the reference coefficients (ground-truth) for assessing the coefficient estimation quality of the compared methods.

**Table 1:** Eight simulation settings that are evaluated for each method. The sub-scripted triangle (△) is used to indicate high collinearity settings. The star (*) refers to the real-data version of a simulated setting

Setting	*y*	*N*	No. predictors	EPV	Median VIF
*A*/A*	Xerostomia	592	7	23	5
*A*_△_	Xerostomia	592	7	23	43
*B*	Xerostomia	592	19	8	5
*B*_△_/*B*_△_*	Xerostomia	592	19	8	43
C/C*	Dysphagia	592	13	6	7
*C*_△_	Dysphagia	592	13	6	43
*D*	Dysphagia	592	43	2	7
*D*_△_/*D*_△_*	Dysphagia	592	43	2	43

To study the effect of collinearity independently of the number of predictors and the number of events-per-variable (EPV), we generated another four simulation settings: for each setting with a large predictor set that inherently exhibits high collinearity (*B*_△_ and *D*_△_) we generate^3^ low-collin earity variants (*B* and *D* respectively), and for each setting with a small predictor set that inherently exhibits a lower degree of collinearity (*A* and *C*) we generate high-collinearity variants (*A*_△_ and *C*_△_ respectively). Finally, we end up with a total of eight simulation settings, for which four pair-wise comparisons can be made to assess the effect of collinearity.

To assess to what degree the simulation is accurate for the actual clinical prediction modeling problem, we compare the results of the simulation to a comparable real-data setting. These real-data experiments are indicated by a star (∗) in Table 1 and have the same modeling characteristics as the corresponding simul ations: the same predictor covariance, outcome prevalence, and sample size [32].

#### Estimators/target of analysis

We quantify collinearity by the median variance inflation factor (VIF). The VIF of a predictor reflects the relative increase in coefficient variance for that predictor due to the presence of other predictors. A VIF of 1 indicates absence of collinearity, whereas a VIF larger than 10 is often considered to reflect a high degree of collinearity [33].

#### Application of the methods

Besides standard logistic regression (LR), we compare all methods discussed in the previous section: Lasso, Ridge, ElasticNet, PCLR, LAELR, Dropout, and LR_NN_ (the use of non-negativity constraints for dosage coefficients through gradient projection). These are listed in Table 2.

**Table 2:** List of compared methods

Method	Abbreviation	Hyperparameters
Logistic regression	LR	–
Lasso penalization	Lasso	λ_ℓ1_ (penalty importance)
Ridge penalization	Ridge	λ_ℓ2_ (penalty importance)
Elastic Net penalization	ElasticNet	λ_ℓ1_, λ_ℓ2_ (importance per penalty)
Dropout regularization	Dropout	δ (dropout ratio)
Principal component logistic regression	PCLR	*d*_PCA_ (number of components)
Linear auto-encoder logistic regression	LAELR	*d*_LAE_ (number of components) λ_LAE_ (importance of reconstruction loss)
Non-negative logistic regression	LR_NN_	–

For a fair comparison, we perform equal hyperparameter^4^ tuning across methods. For all models, we tune hyperparameters using Bayesian optimization [34] in a (nested) 3-fold cross-validation setting, with a log-likelihood tuning criterion. As general data preprocessing we standardize all predictors to have zero-mean and unit variance. More details about the exact training criteria for each method, hyperparameter tuning, and optimization [35–37] can be found in Additional file 3.

#### Performance measures

We analyze our aims with regard to the measures stated in Table 3. We use four measures to evaluate different aspects of prediction model performance: the area under the receiver-operator characteristic curve (AUC) measures how well the prediction model ranks patients based on their predicted risks in relation to the observed outcomes. An AUC of 1 indicates that patients with the outcome can be perfectly separated from patients without the outcome based on their predicted risk, whereas an AUC of 0.5 indicates that the ranking of patients is arbitrary regarding their observed outcomes. Calibration intercept (Intercept) quantifies how well the mean predicted risk corresponds to the overall observed outcome prevalence. An Intercept of 0 indicates perfect correspondence between mean predicted risk and the observed outcome prevalence, while negative Intercept values indicate general overestimation, and positive Intercept values a general underestimation of predicted risk. Calibration slope (CS) evaluates the extremity of the predicted risks, with a CS below 1 indicating too extreme predictions (high-risk patients receive too high predicted risks and low-risk patients receive too low predicted risks), while CS above 1 indicate too conservative predictions (biased towards the mean predicted risk). Nagelkerke *R*^2^ measures more generally how much of the variation in the observed outcomes can be explained by the model’s predictions. To measure the quality of the estimated coefficients, we calculate their mean absolute error (MAE) with the ground-truth coefficients. A less explored measure we use is the expected proportion of included coefficients that has the same direction of effect (positive, negative, or zero) across two simulated model construction repetitions (the mean Jaccard index of the coefficient signs: MJICS, ranging from 0 to 1). This measure is formally defined in Eq. 2, for arbitrary samples *i* and *j*, to assess the robustness of the coefficient interpretation when developing a prediction model: we consider methods that include^5^ the same predictors in the model and assign the same directions of effect when repeating the model construction process to be more robust than methods that include different coefficients or assign different direction of effect across iterations.

1 $$ \mathrm{MJICS}=\frac{\left|\operatorname{sgn}\left(\hat{{\boldsymbol{\theta}}_{\boldsymbol{i}}}\right)\cap \operatorname{sgn}\left(\hat{{\boldsymbol{\theta}}_{\boldsymbol{j}}}\right)\right|}{\left|\operatorname{sgn}\left(\hat{{\boldsymbol{\theta}}_{\boldsymbol{i}}}\right)\cup \operatorname{sgn}\left(\hat{{\boldsymbol{\theta}}_{\boldsymbol{j}}}\right)\right|} $$

2 $$ \mathit{\operatorname{sgn}}(x)=\left\{\begin{array}{rr}-1& iff\kern0.5em x<-0.01\\ {}1& iff\kern0.5em x>0.01\\ {}0& otherwise\end{array}\right. $$

**Table 3:** Overview of the measures used to compare methods on predictive performance and coefficient estimation

Measure	Abbreviation	Ideal value
Predictive performance		
Area under the receiver-operator characteristic curve	AUC	1
Calibration intercept	Intercept	0
Calibration slope	Slope	1
Nagelkerke *R*-squared	*R*^2^	1
Coefficient estimation		
Mean absolute error between the estimated and the true coefficients	MAE	0
Mean proportion of coefficients with the same direction of effect after repetition	MJICS	1

All measures are estimated by repeatedly sampling (*n*_rep._ = 100) a new dataset from the constructed Gaussian distributions, refitting all models in each dataset, and evaluating them in a validation set generated from the same distributions as the development set of size *N* = 10,000. The reported 95% confidence intervals are based on these repetitions, and reflect variability of the entire model construction procedure: sampling training data, developing the model (including hyperparameter tuning), and sampling a new validation set. For the real-data settings, a repeated 5-fold cross validation (*N* = 592 per fold) on the real data is used to estimate each measure, and their respective confidence intervals (*n*_rep._ = 100) [38].

#### Coding and execution

All experiments were implemented in Python 3.6, primarily using Scikit-learn [39] and PyTorch [40]. Predictive performance measures are calculated in R 3.6.1, using the val.prob.ci.2 function [41]. The computer code used to conduct the experiments is available at https://github.com/tuur/collinearity. The original patient data is not available for patient privacy reasons. 

## Results

This section presents the simulation results with regard to predictive performance and coefficient estimation. Based on a comparison between our simulations and the real-data experiments in terms of predictive performance we concluded that the simulations are in accordance with the real-data settings. Results of the real-data experiments can be found in Additional file 1.

### Predictive performance

**Fig. 1:**
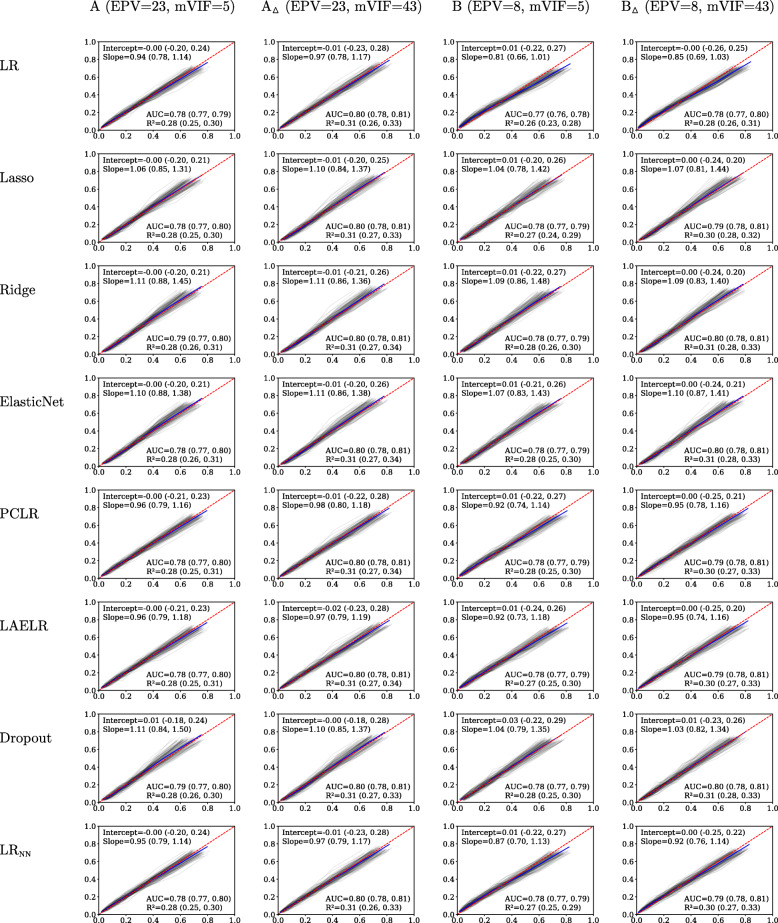
Predictive performance results for the xerostomia simulations. Lowess-smoothed calibration curves per simulation are plotted in grey. The calibration curve over all repetitions is shown in blue. Perfect calibration, the diagonal, is dashed in red

**Fig. 2:**
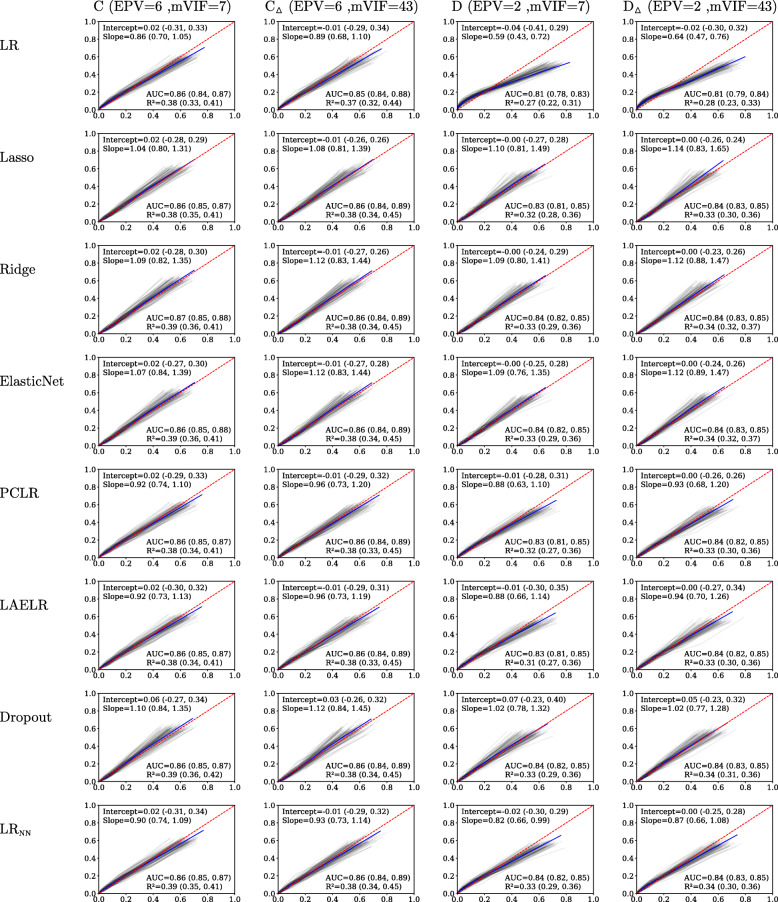
Predictive performance results for the dysphagia simulations. Lowess-smoothed calibration curves per simulation are plotted in grey. The calibration curve over all repetitions is shown in blue. Perfect calibration, the diagonal, is dashed in red

Simulation results regarding calibration and discrimination are reported in Fig. 1 for the xerostomia settings and in Fig. 2 for the dysphagia settings. We observed no effects of collinearity on the predictive performance of any of the compared methods: in terms of AUC, *R*^2^, Intercept, Slope, nor the calibration plots (comparing *A* with *A*_△_, and *B* with *B*_△_). Based on the calibration plots in Fig. 1, we do observe a slight overall overestimation of risk for LR compared to the other methods when extending the predictor set (comparing *B* to *A*, and *B*_△_ to *A*_△_), probably due to the lower EPV.

We obtained similar results for the simulated dysphagia settings, finding no effect (AUC, *R*^2^, Intercept, Slope) of collinearity on predictive performance, and little to no difference between the compared methods in any of the performance measures (AUC, *R*^2^, Intercept, Slope). Again, LR yielded worse calibration compared to the other methods (irrespective of the degree of collinearity). As expected, the difference between LR and the other compared methods was largest in terms of both calibration and discrimination in the setting with the lowest EPV (setting D, with an EPV of 2), indicating that LR suffers most from overfitting.

### Coefficient estimation

**Fig. 3:**
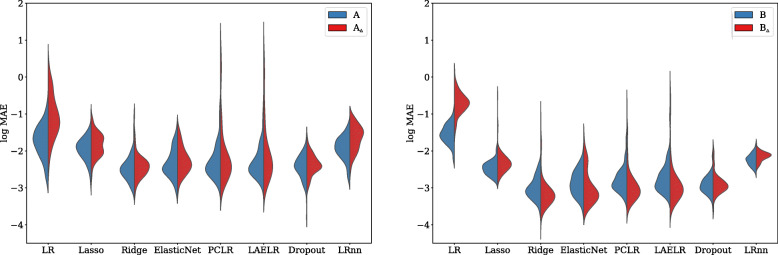
Across models, the log mean absolute error between the estimated and the true coefficients for each method, for the xerostomia settings. Red indicates high collinearity, and blue low collinearity.

**Fig. 4:**
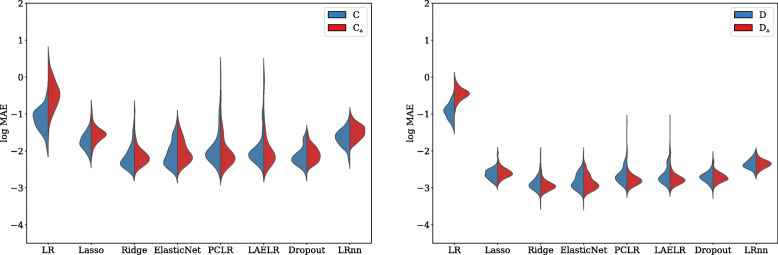
Across models, the log mean absolute error between the estimated and the true coefficients for each method, for the dysphagia settings. Red indicates high collinearity, and blue low collinearity

Observing the estimation of the regression coefficients shown in Figs. 3 and 4, we found that in terms of MAE between the estimated coefficients and the true coefficients, in both the higher and lower collinearity settings LR had a higher MAE, followed by LR_NN_, in turn followed by Lasso^6^. Ridge, ElasticNet, PCLR, LAELR, and Dropout had lower MAE than LR, LR_NN_, and Lasso, but did not differ among one another. Regarding the effect of collinearity, LR was the only method that showed a higher MAE in the high collinearity settings, compared to the lower collinearity settings.

**Fig. 5:**
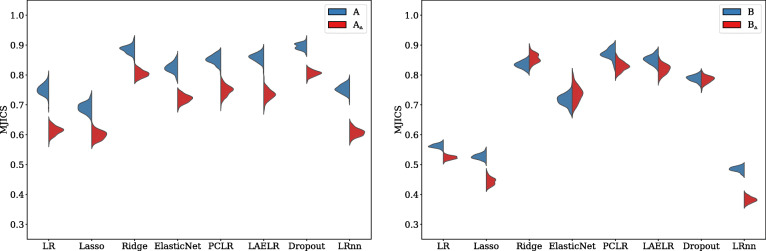
Across models, the mean proportion of coefficients with the same direction of effect after repetition for the xerostomia settings. Red indicates high collinearity, and blue low collinearity

**Fig. 6:**
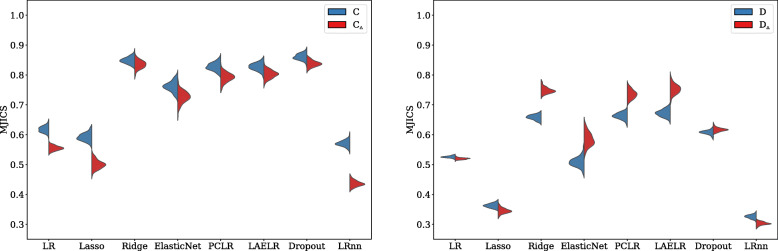
Across models, the mean proportion of coefficients with the same direction of effect after repetition for the dysphagia settings. Red indicates high collinearity, and blue low collinearity

When observing the stability of the predictor selection (to what degree the same predictors were selected with the same directions of effect when repeating the model development process across simulations), observing Figs. 5 and 6, we found that in all settings, LR_NN_ had less stable predictor selection than the other methods, followed by Lasso, LR, and ElasticNet. Ridge, Dropout, LAELR, and PCLR had more stable predictor selection than the formerly mentioned methods but showed no consistent ranking among each other.

Regarding the effect of collinearity, LR_NN_, Lasso, and LR were the only methods that showed a decrease in stability of predictor selection with an increase in collinearity across all settings. In settings *A* and *C*, we did find a negative effect of collinearity on selection stability also for the other methods. However, in settings *B* and *D*, we found even an increase in stability of predictor selection for Ridge and ElasticNet, but no consistent effect for Dropout, LAELR, and PCLR. Based on these results, we conjecture that the effect of collinearity may be explained by two aspects. First, collinearity negatively affects the stability of maximum likelihood-based coefficient selection (reducing MJICS), due to the increased variance in coefficient estimation. This can explain why the negative effect remains present for LR and LR_NN_ across all settings: coefficient estimation for these methods is purely likelihood based.

**Fig. 7:**
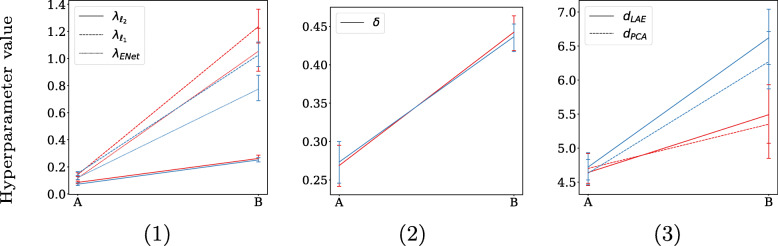
Hyperparameter values for xerostomia: per predictor set, setting *A* being the small predictor set with high EPV (EPV = 23), and setting *B* the large predictor set with lower EPV (EPV = 8). The high collinearity settings in red, and the low collinearity setting in blue. The methods are distributed across three plots due to their different scales. Hyperparameter notation follows Table 2, except for λ_ENet_ , which is the total shrinkage factor for ElasticNet (λ_ℓ1_ +λ_ℓ2_ )

**Fig. 8:**
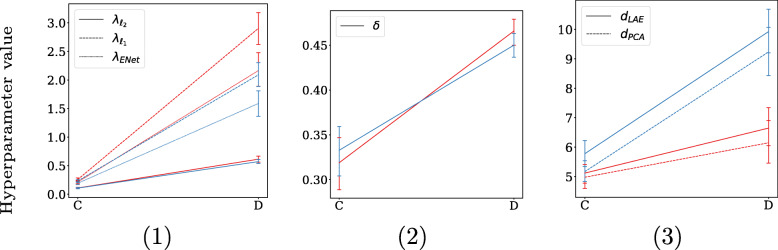
Hyperparameter values for xerostomia: per predictor set, setting *C* being the small predictor set with high EPV (EPV = 6), and setting *D* the large predictor set with lower EPV (EPV = 2). The high collinearity settings in red, and the low collinearity setting in blue. The methods are distributed across three plots due to their different scales. Hyperparameter notation follows Table 2, except for λ_ENet_ , which is the total shrinkage factor for ElasticNet (λ_ℓ1_ + λ_ℓ2_)

The second aspect is that of regularization, which can—for some methods—have a stabilizing effect of coefficient selection. The degree of regularization is determined by the hyperparameter tuning process, which is indirectly impacted by the EPV: low EPV settings are more likely to result in overfitting, and consequently obtain a larger degree of regularization. High EPV settings are less prone to overfitting and consequently obtain less regularization. By observing the used degree of regularization by each method in Figs. 7 and 8, it can be noticed that less regularization is used in the low EPV settings *A* and *C*, and coefficient estimation is more driven by maximum likelihood compared to their high EPV counterparts *B* and *D*^7^.

Ridge, Dropout, and ElasticNet all quadratically penalize coefficient size, resulting in a grouping effect of collinear predictors. When regularization is strong, and collinearity is high, this constitutes a strong grouping effect, which in turn stimulates stable predictor selection.

For PCLR and LAELR, a larger degree of regularization implies a heavier dependence on the principal components that explain the variance among predictors. As collinearity increases, a smaller number of components is required to explain the same amount of variance among predictors. This can be directly observed in Figs. 7(3) and 8(3), where for the large predictor sets (B and D) hyperparameter tuning resulted in a smaller number of components for PCLR and LAELR when collinearity was higher. This reliance on less components can in turn result in more stable coefficient estimation.

For Lasso, and partially ElasticNet, more regularization implies a stronger predictor selection effect, resulting in smaller models. Stronger selection in itself decreases the likelihood of (by chance) selecting the same coefficients when developing the model on a different sample. We conjecture that this is the reason why Lasso and LR_NN_ have low overall MJICS, independently of collinearity compared to the other methods. Additionally, as Lasso’s selection is likelihood-based, the negative impact of collinearity on predictor selection, as observed for LR and LR_NN_, also affects Lasso. This can be observed by the reduction of MJICS in the high collinearity settings in Figs. 5 and 6.

## Discussion

The current study investigated the effect of collinearity on predictive performance and the stability of coefficient estimation, comparing eight different methods in a simulation study on the construction of prediction models that estimate the risk on complications after radiotherapy in head-and-neck cancer patients.

In this paper, we found little to no impact of collinearity on predictive performance (discrimination and calibration of the fitted models) across methods and simulation settings. For standard logistic regression, and methods that have a strong predictor selection effect (Lasso, and non-negative logistic regression) the stability of predictor selection was generally lower compared to other methods, and was negatively influenced by collinearity across all simulations. We observed that, although in high-EPV settings collinearity had a negative effect on the stability of predictor selection across all methods, in the lower-EPV settings, that consequently required a larger amount of regularization, the negative impact of collinearity on predictor selection stability was smaller for methods that distribute the explained outcome variance more evenly across collinear predictors (Ridge, ElasticNet, Dropout, PCLR, and LAELR).

Harrell et al. [8] mentioned that when there is no difference in the degree of collinearity between development and validation data, collinearity is generally not considered a problem for predictive performance, but can be problematic for reliable variable selection when performing stepwise selection. This was also confirmed by Cohen et al. [42], and later also by Dormann et al. [43], who compared 23 methods (including various dimensionality reduction techniques and shrinkage-based methods) to address collinearity in five simulated ecological predictor-response relationships. The current study findings are in line with these earlier works and provide additional evidence to support this. An important note to make is that in low-dimensional settings (where the number of predictors is smaller than the number of samples) with correlating predictors earlier work by Tibshirani et al. [11], Zou et al. [13], and Pavlou et al. [44] empirically found that selection-based approaches like Lasso yielded lower predictive performance compared to for example Ridge. The current study did not find such a difference in predictive performance between Lasso and Ridge in any of the eight settings.

Nevertheless, for addressing collinearity in clinical prediction models, we would recommend refraining from data-driven predictor selection approaches (like Lasso), because of the increased instability of predictor selection in the presence of collinearity, even in relatively high EPV settings. Even though the individual coefficient values are generally not of primary interest in prediction modeling studies, the selection of certain predictors over others may disproportionally give the impression that included predictors have a stronger association with the outcome than excluded predictors (e.g., when performing face validity checks by physicians with a model in which some clinically viable predictors have been excluded due to collinearity).

There are several limitations that should be considered when interpreting this study. Firstly, the current work has focused only on low-dimensional settings and binary logistic regression models. Future studies may evaluate the effect of collinearity, for instance in settings with multiple outcomes (e.g., multi-nomial regression). Finally, we focused on evaluation of predictive performance in the same population, under no change of collinearity structure between the development and validation data. Collinearity has been shown to have a negative impact on performance under changes between development and validation data, and is considered a difficult challenge to overcome, for which a good understanding of the underlying mechanism causing the collinearity is crucial [43].

We believe that beside being able to anticipate how harmful a change in collinearity between development and validation data may be for predictive performance, an interesting direction of future research is to study how background knowledge about the underlying collinearity mechanism, can be used to adapt prediction models accordingly.

## Conclusions

When prediction models are developed on data with high correlations among predictors, model coefficients may inhibit large variance, possibly resulting in unexpected predictor-outcome associations. Comparing a range of methods to address such variance in a simulation study showed that the choice of method had little impact on predictive performance. Nevertheless, methods performing strong predictor selection (e.g., Lasso) showed increased instability in their selection when collinearity was high, even in relatively high events-per-variable settings, while predictor selection stability of certain other methods (Ridge, PCLR, LAELR, and Dropout) was more robust against changes in collinearity. Therefore, we recommend the use of Ridge, PCLR, LAELR, or Dropout over the use of data-driven predictor selection approaches in high-collinearity settings, as resulting models may disproportionally give the impression that included predictors have a stronger association with the outcome than excluded predictors.

## Supplementary Information

**Additional file 1.** Results on the real study data.

**Additional file 2.** Real study data characteristics and specification of predictors.

**Additional file 3.** Detailed description of each compared method.

**Additional file 4.** Correlation plots per predictor set.

**Additional file 5.** Obtained model coefficients per experimental setting, including the real data settings.

**Additional file 6.** Coefficient estimation results using mean squared error.

## Data Availability

The computer code (Python 3.6 and R 3.6.1) used to conduct the experiments is available at https://github.com/tuur/collinearity. The original patient data is not available, for patient privacy reasons.

## Abbreviations

PCA: Principal component analysis
PCLR: Principal component logistic regression
LAE: Linear auto-encoders
LAELR: Linear autoencoder logistic regression
NN: Non-negativity
NTCP: Normal-tissue complication probability
OAR: Organ at risk
ADEMP: Aims, data-generating mechanisms, estimands, methods, and performance measures
UMCG: University Medical Center Groningen
EPV: Events per variable
VIF: Variance inflation factor
LR: Logistic regression
MJICS: Mean Jaccard index of the coefficient signs
AUC: Area under the receiver-operator characteristic curve
Intercept: Calibration intercept
Slope: Calibration slope
*R*^2^: Nagelkerke *R*-squared
MAE: Mean absolute error between the estimated and the true coefficients

## Footnotes

1 More detailed descriptions of the used predictor sets are given in Additional file 2.

2 In a 5-fold cross validation on the real data, Ridge yielded good results in terms of calibration and discrimination, but also included the largest proportion OAR in the model (relevant for this case study).

3 We change the degree of collinearity by scaling the covariance matrix of the multi-variate Gaussian (without changing the diagonal). In doing so, the degree of class separation and the outcome prevalence of the ground-truth model may change. To maintain the same degree of class separation in the data, we scale the slope of the ground-truth model. Additionally, to maintain the same outcome prevalence, we adjusted the ground-truth intercept accordingly. This way, we change the degree of collinearity, but maintain ground-truth area under the receiver operator curve, and outcome prevalence.

4 Parameters that are not part of the model itself, but steer how the coefficients are determined (e.g., the relative importance of the shrinkage penalty in Lasso and Ridge, or the number of components for PCLR, among others).

5 The maximum likelihood estimation procedure, for all models, rarely estimated coefficients to be exactly 0. Considering its implementation using 32-bit floats, returning 0 means 0.0000000, up to the 7th decimal. By choosing the pragmatic threshold of 0.01 we aimed to discriminate coefficients that positively contribute to risk (> 0.01), coefficients having no practical contribution to risk (between − 0.01 and 0.01), or coefficients having a negative contribution to risk (< − 0.01). This way, we discriminate between coefficients of e.g., 0.3 and 0.000001, as we believe the latter could in practice be considered excluded.

6 We also analyzed these results us regarding mean squared error (MSE) and found similar findings as for MAE. These results can be found in [Additional file 6].

7 Notice that λ_ℓ1_, λ_ℓ2_, and λ_ENet_ are shrinkage factors: higher values indicate a larger degree of regularization. A larger degree of dropout ratio δ indicates a larger degree of regularization. With regard to PCLR and LAELR it is important to notice that the number of components is only indicative of the degree of regularization within the same predictor set: In Figs. 7(3) and 8(3), the larger number of components in settings B and D in comparison to settings A and C do not imply less regularization, as the original dimensions of B and D are in itself much larger than that of A and C (19 and 43 compared to 7 and 13).
