# Supplementary figures and images for: Performance of binary prediction models in high-correlation low-dimensional settings: a comparison of methods
Source: Diagn Progn Res. 2022 Jan 11;6:1. doi: 10.1186/s41512-021-00115-5 (PMC8751246; doi:10.1186/s41512-021-00115-5)

# Additional file 1

## Results on the real study data.

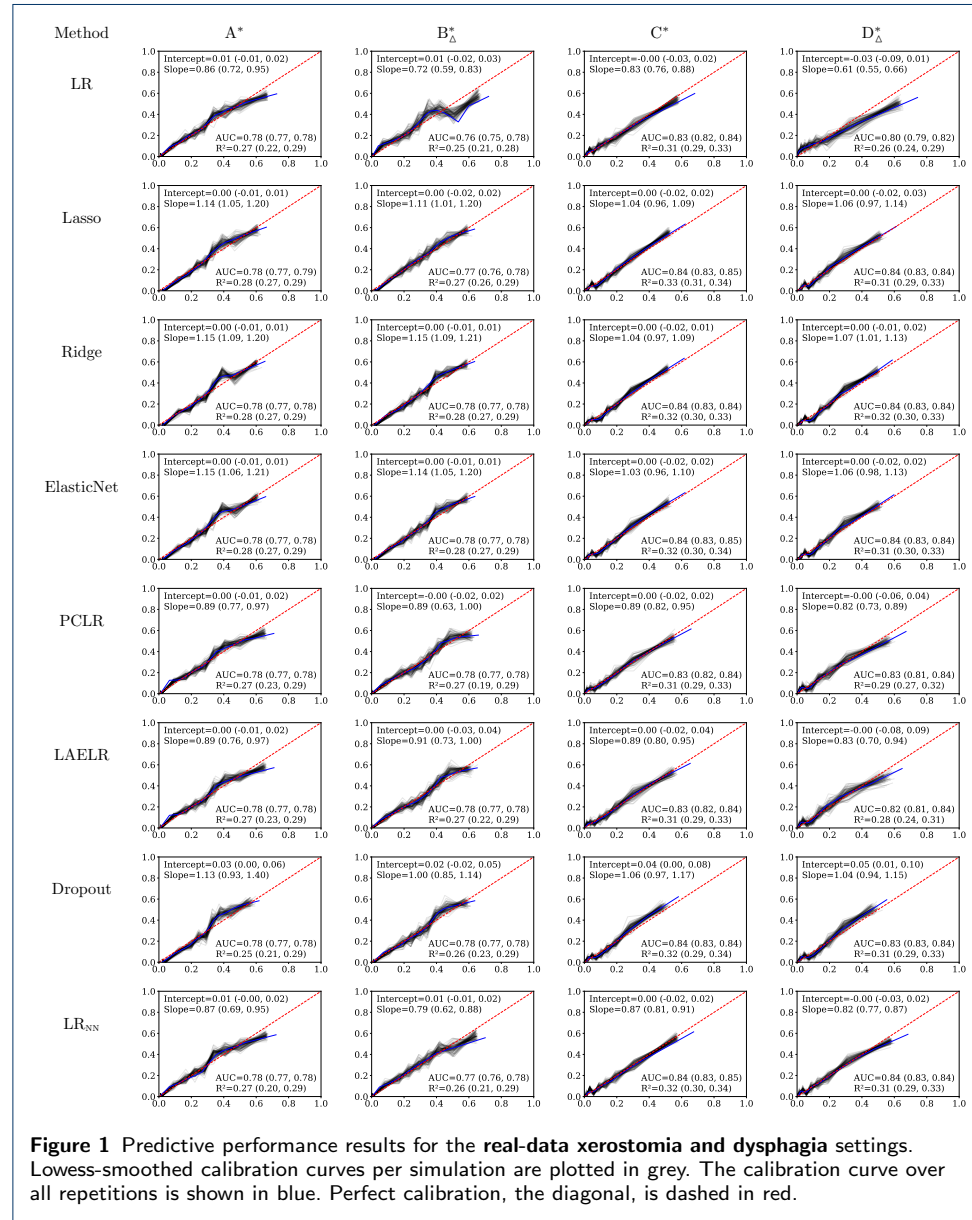

Supplement: Supplementary file 1 — Additional file 1. Results on the real study data. [file 41512_2021_115_MOESM1_ESM.pdf]

# Additional file 6

## Coefficient estimation results using mean squared error.

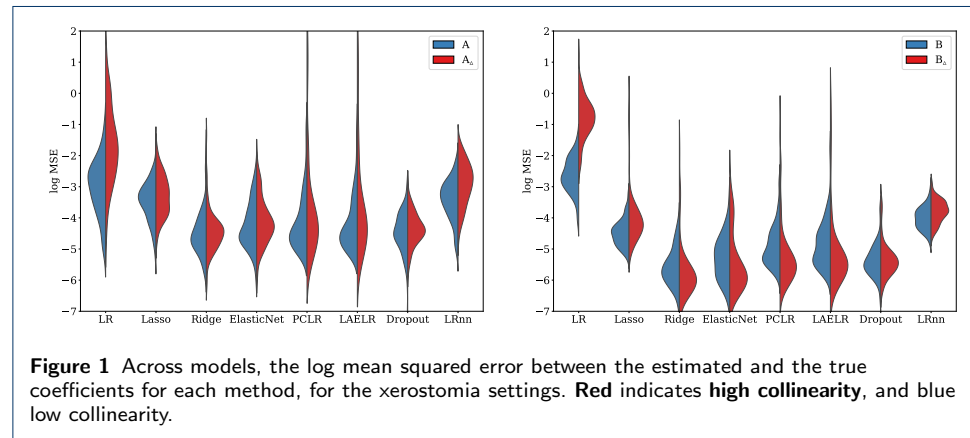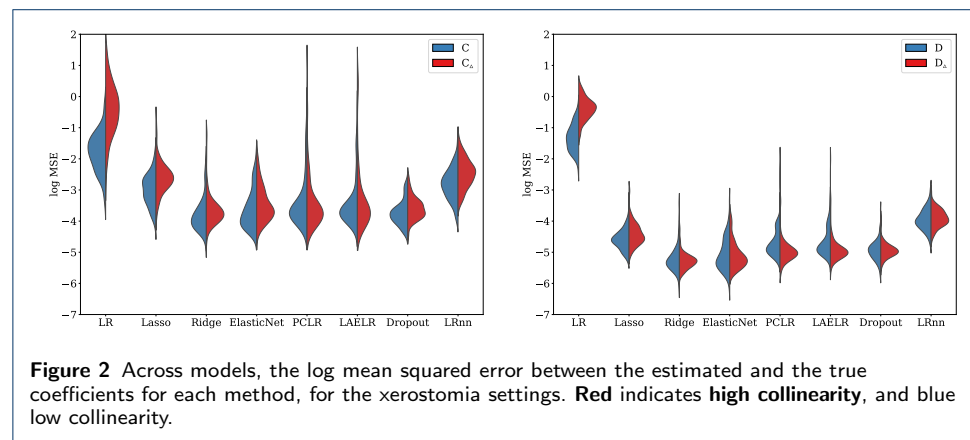

Supplement: Supplementary file 6 — Additional file 6. Coefficient estimation results using mean squared error. [file 41512_2021_115_MOESM6_ESM.pdf]
