# Supplementary material for: Performance of binary prediction models in high-correlation low-dimensional settings: a comparison of methods
Source: Diagn Progn Res. 2022 Jan 11;6:1. doi: 10.1186/s41512-021-00115-5 (PMC8751246; doi:10.1186/s41512-021-00115-5)
Supplement: Supplementary file 2 — Additional file 2. Real study data characteristics and specification of predictors. [file 41512_2021_115_MOESM2_ESM.pdf]

Additional file 2

Real study data characteristics and specification of predictors.

Table 1 General study population characteristics. For age we report the median and standard deviation. For the other variables, we report the number and the percentage of the whole population.

	**UMCG** (n=740)
**Patient characteristics (at baseline)**	
Age	63 (10)
Gender	
Men	552 (75%)
Women	188 (25%)
Primary tumor site	
Pharynx	356 (48%)
Larynx	344 (46%)
Oral cavity	40 (5%)
Treatment modality	
Conventional radiotherapy	159 (21%)
Accelerated radiotherapy	287 (39%)
Chemoradiation	252 (34%)
Bioradiation	42 (6%)
T-classification	
Tis-T1	136 (18%)
T2	246 (33%)
T3	182 (25%)
T4	176 (24%)
N-classification	
N0	356 (48%)
N1	64 (9%)
N2	303 (41%)
N3	17 (2%)
**Predicted outcomes (at M6)**	
Xerostomia $\geq$ Grade 2	200 (27%)
Dysphagia $\geq$ Grade 2	102 (14%)

Table 2 Predictors used in each predictor set. $\mathbb{R}$ refers to continuous predictors, and $\{0,1\}$ refers to binary predictors. PCM refers to the pharyngeal constrictor muscle. Organs at risk were delineated following consensus guidelines [1]. Dose is expressed in Gray (Gy).

**Predictor**	A^* (dim=7)	B^*_Δ (dim=19)	C^* (dim=13)	D^*_Δ (dim=43)
Age	$\mathbb{R}$	$\mathbb{R}$	$\mathbb{R}$	$\mathbb{R}$
Grade 1 complications at baseline	$\{0,1\}$	$\{0,1\}$	$\{0,1\}$	$\{0,1\}$
Grade 2 complications at baseline	$\{0,1\}$	$\{0,1\}$	$\{0,1\}$	$\{0,1\}$
Submandibular left: mean dose	$\mathbb{R}$	$\mathbb{R}$	$\mathbb{R}$	$\mathbb{R}$
Submandibular left: V10, V30, V50	-	$\mathbb{R}$	-	$\mathbb{R}$
Submandibular right: mean dose	$\mathbb{R}$	$\mathbb{R}$	$\mathbb{R}$	$\mathbb{R}$
Submandibular right: V10, V30, V50	-	$\mathbb{R}$	-	$\mathbb{R}$
Parotid left: mean dose	$\mathbb{R}$	$\mathbb{R}$	$\mathbb{R}$	$\mathbb{R}$
Parotid left: V10, V30, V50	-	$\mathbb{R}$	-	$\mathbb{R}$
Parotid right: mean dose	$\mathbb{R}$	$\mathbb{R}$	$\mathbb{R}$	$\mathbb{R}$
Parotid right: V10, V30, V50	-	$\mathbb{R}$	-	$\mathbb{R}$
PCM.Superior.Dm	-	-	$\mathbb{R}$	$\mathbb{R}$
PCM.Superior V10, 30, 50	-	-	-	$\mathbb{R}$
PCM.Med.Dm	-	-	$\mathbb{R}$	$\mathbb{R}$
PCM.Med V10, 30, 50	-	-	-	$\mathbb{R}$
PCM.Inferior.Dm	-	-	$\mathbb{R}$	$\mathbb{R}$
PCM.Inferior V10, 30, 50	-	-	-	$\mathbb{R}$
Supraglottic.Dm	-	-	$\mathbb{R}$	$\mathbb{R}$
Supraglottic V10, 30, 50	-	-	-	$\mathbb{R}$
OralCavity.Ext.Dm	-	-	$\mathbb{R}$	$\mathbb{R}$
OralCavity.Ext. V10, 30, 50	-	-	-	$\mathbb{R}$
GlotticArea.Dm	-	-	$\mathbb{R}$	$\mathbb{R}$
GlotticArea V10, 30, 50	-	-	-	$\mathbb{R}$

References

1. Brouwer CL, Steenbakkers RJ, Bourhis J, Budach W, Grau C, Grégoire V, et al. CT-based delineation of organs at risk in the head and neck region: DAHANCA, EORTC, GORTEC, HKNPCSG, NCIC CTG, NCRI, NRG Oncology and TROG consensus guidelines. Radiotherapy and Oncology. 2015;117(1):83–90.
