# Supplementary material for: Performance of binary prediction models in high-correlation low-dimensional settings: a comparison of methods
Source: Diagn Progn Res. 2022 Jan 11;6:1. doi: 10.1186/s41512-021-00115-5 (PMC8751246; doi:10.1186/s41512-021-00115-5)
Supplement: Supplementary file 3 — Additional file 3. Detailed description of each compared method. [file 41512_2021_115_MOESM3_ESM.pdf]

Additional file 3

Detailed description of each compared method.

In this section we provide a concise but more precise description of each method used in the experiments of this paper, to facilitate easier reproducibility.

The data

In this appendix, we use matrix $\boldsymbol{X} = \langle \boldsymbol{x}_1, \boldsymbol{x}_2, ..., \boldsymbol{x}_n \rangle$ of dimension $n \times d$ to describe the n feature vectors representing the n patients. Each feature vector $\boldsymbol{x}_i$ is of dimension d. The outcome vector $\boldsymbol{y}$ is used to denote the n outcome labels (one or zero), where y_i is the value at dimension i of y, corresponding to the observed ("ground truth") outcome of $\boldsymbol{x}_i$.

The link function

With the term *link function*, we refer to the (parameterized) function that maps the predictor values to predicted outcome probability. For all methods, the link function used to calculate predicted outcome $\hat{y}_i$ from input x_i is the logit function:

$$f(\boldsymbol{x}_i, \boldsymbol{\beta}, \beta_0) = \frac{1}{\exp\{-(\boldsymbol{\beta}\boldsymbol{x}_i + \beta_0)\}}, \tag{1}$$

where $\boldsymbol{\beta} = \langle \beta_1, \beta_2, ..., \beta_d \rangle$ is the vector of coefficients, and β_0 the intercept.

The objective function

The *objective function* defines what are considered *optimal* coefficients for each method (in some literature referred to as the training criterion). A method's objective function is generally defined to return coefficients $\boldsymbol{\beta}$ and intercept β_0 that minimize the training error, which is given by a *loss function* (in some literature referred to as the cost function). Since all methods in this study have logistic regression form, the only difference between the compared methods is how coefficients are obtained from the data, i.e., they differ only in their objective function. The component of the objective function that is shared by all methods is the cross-entropy loss (i.e., the negative log-likelihood):

$$L_{\mathrm{ML}}(\boldsymbol{\beta}, \beta_0) = -\sum_{i=1}^{n} y_i \log f(\boldsymbol{x}_i, \boldsymbol{\beta}, \beta_0) + (1 - y_i) \log(1 - f(\boldsymbol{x}_i, \boldsymbol{\beta}, \beta_0)) \tag{2}$$

For our baseline model, regular logistic regression, the corresponding objective consists purely of finding coefficient values β^* and intercept value β_0^* that minimize the cross-entropy (equivalent to maximum likelihood):

$$\boldsymbol{\beta}^*, \beta_0^* = \underset{\boldsymbol{\beta}, \beta_0}{\arg\min}\, L_{\mathrm{ML}}(\boldsymbol{\beta}, \beta_0) \tag{3}$$

In the sections below, we will specify for each method how the corresponding objective differs.

Loss minimization and hyperparameter tuning

Unless specified otherwise, each method's loss function is minimized using Adam [1], for at most 1000 epochs, using early stopping [2] with a patience of 500 epochs, and a maximum learning rate of 0.1. For tuning of important hyperparameters that may come with certain methods, we use nested cross-validation, in a Bayesian optimization setting [3], using 10 iterations of a Gaussian process with a certain prior and range. The range and prior of each hyperparameter are given in the sections below.

Lasso

For Lasso, the only difference with regard to the baseline is an extension of the $L_{\mathrm{ML}}(\boldsymbol{\beta}, \beta_0)$ with a penalty on the size of the coefficient values, the ℓ_1-norm of the coefficients, shown in Equation 4, where λ_{ℓ_1} is a hyperparameter determining the importance of the ℓ_1-penalty.

$$L_{\ell_1} = L_{\mathrm{ML}}(\boldsymbol{\beta}, \beta_0) + \lambda_{\ell_1} |\boldsymbol{\beta}| \tag{4}$$

$$\boldsymbol{\beta}^*, \beta_0^* = \underset{\boldsymbol{\beta}, \beta_0}{\arg\min}\, L_{\ell_1} \tag{5}$$

The tuning range for λ_{ℓ_1} is $[10^{-2}, 10^3]$, and we used a log-linear prior. The final objective is shown in Equation 5.

Ridge

For Ridge, the modification with regard to the baseline is very similar as for Lasso, except that the penalty on coefficient size is the ℓ_2-norm of the $\boldsymbol{\beta}$. This results in loss function, and objective functions 6, and 7 respectively.

$$L_{\ell_2} = L_{\mathrm{ML}}(\boldsymbol{\beta}, \beta_0) + \lambda_{\ell_2} |\boldsymbol{\beta}|^2 \tag{6}$$

$$\boldsymbol{\beta}^*, \beta_0^* = \underset{\boldsymbol{\beta}, \beta_0}{\arg\min}\, L_{\ell_2} \tag{7}$$

For Ridge, the hyperparameter λ_{ℓ_2} is tuned with the same range and prior as λ_{ℓ_1} for Lasso.

Elastic Net

Elastic Net is the application of both a Lasso and a Ridge penalty to the model. Consequently, the final loss function, and objective function of Elastic Net can be given by Equations 8, and 9 respectively.

$$L_{\mathrm{ENet}} = L_{\mathrm{ML}}(\boldsymbol{\beta}, \beta_0) + \lambda_{\ell_1} |\boldsymbol{\beta}| + \lambda_{\ell_2} |\boldsymbol{\beta}|^2 \tag{8}$$

$$\boldsymbol{\beta}^*, \beta_0^* = \underset{\boldsymbol{\beta}, \beta_0}{\arg\min}\, L_{\mathrm{ENet}} \tag{9}$$

The tuning procedure of hyperparameters λ_{ℓ_1} and λ_{ℓ_2} is the same as for Lasso and Ridge.

Principal Component Logistic Regression (PLCR)
In PCLR, the input matrix $\boldsymbol{X}$ is first projected into its principal components, using principal component analysis (PCA), before applying (logistic) regression. PCA aims to find a linear projection $\boldsymbol{W}^*$ that maps $\boldsymbol{X}$ to a (smaller) set of non-correlating latent variables $\boldsymbol{H} := \boldsymbol{W}^*\boldsymbol{X}$ (the principal components) that best capture the variance in X. The loss function, and objective to find projection $\boldsymbol{W}^*$ are given by Equations 10 and 11 respectively [4]. The number of latent variables (or principal components) is tuned using a linear prior over integer values in $[4, d]$.

$$L_{\text{PCA}}(\boldsymbol{W}) = |\boldsymbol{X} - \boldsymbol{W}^\top \boldsymbol{W}\boldsymbol{X}|^2 \quad (10)$$

$$\boldsymbol{W}^* = \arg\min_{\boldsymbol{W}} L_{\text{PCA}}(\boldsymbol{W}) \quad (11)$$

$$\text{subject to } \boldsymbol{W}^\top \boldsymbol{W} = \boldsymbol{I}$$

After projecting each input vector $\boldsymbol{x}_i \in \boldsymbol{X}$ to its latent vector $\boldsymbol{h}_i \in \boldsymbol{H}$, a logistic regression $g(\boldsymbol{h}_i, \boldsymbol{\gamma}, \gamma_0)$ is fitted to relate the latent variables $\boldsymbol{H}$ to the outcome $\boldsymbol{Y}$, using coefficients $\boldsymbol{\gamma}$, and intercept γ_0, following standard maximum likelihood optimization (Equation 12).

$$\boldsymbol{\gamma}^*, \gamma_0^* = \arg\min_{\boldsymbol{\gamma}, \gamma_0} L_{\text{ML}}(\boldsymbol{\gamma}, \gamma_0) \quad (12)$$

In this study, we rewrite PCA projection $\boldsymbol{W}^*$, and logistic regression $g(\cdot)$ to an equivalent link function $f(\boldsymbol{x}_i, \boldsymbol{\beta}, \beta_0)$ as in Equation 1 (this is possible as $\boldsymbol{W}^*$ is a *linear* projection), by setting $\boldsymbol{\beta} := \boldsymbol{W}_{1\leq i\leq h}^\top \boldsymbol{\gamma}^*$, and $\beta_0 := \gamma_0^*$. This way $f(\boldsymbol{x}_i, \boldsymbol{\beta}, \beta_0)$ is equivalent to first projecting $\boldsymbol{x}_i$ to latent vector $\boldsymbol{h}_i$, and afterwards obtaining predicted $\hat{y}_i$ using $g(\boldsymbol{h}_i, \boldsymbol{\gamma}, \gamma_0)$. By doing this, PCLR becomes directly comparable in terms of coefficients to the other methods mentioned in this article.

Linear Auto-Encoder Logistic Regression (LAELR)
Linear auto-encoders (LAE) are similar to PCA. The aim of LAE is to find a linear projection $\boldsymbol{W}$ from the input data $\boldsymbol{X}$ to a set of latent variables $\boldsymbol{H}$, that explain the variance in $\boldsymbol{X}$ (in the case of LAE through a linear reconstruction projection $\boldsymbol{V}$). In contrast to PCA, for LAE there is no orthogonality constraint on $\boldsymbol{H}$, and the dimensions of $\boldsymbol{H}$ are not ordered by explained variance. Nevertheless, LAE find projections to the same axis as PCA [5].

$$L_{\text{LAE}}(\boldsymbol{W}, \boldsymbol{V}) = |\boldsymbol{X} - \boldsymbol{V}\boldsymbol{W}\boldsymbol{X}|^2 \quad (13)$$

$$L_{\text{LAELR}}(\boldsymbol{W}, \boldsymbol{V}, \boldsymbol{\gamma}, \gamma_0) = L_{\text{ML}}(\boldsymbol{\gamma}, \gamma_0) + \lambda_{\text{LAE}} L_{\text{LAE}}(\boldsymbol{W}, \boldsymbol{V}) \quad (14)$$

Similar to PCLR, $\boldsymbol{H}$ is related to $\boldsymbol{Y}$ using a logistic regression function $g(\boldsymbol{h}_i, \boldsymbol{\gamma}, \gamma_0)$, and obtain the final coefficients of $f(\boldsymbol{x}_i, \boldsymbol{\beta}, \beta_0)$ by setting $\boldsymbol{\beta} := \boldsymbol{W}_{1\leq i\leq h}^\top \boldsymbol{\gamma}$, and $\beta_0 :=$

γ_0, and the number of latent variables is tuned using a linear prior over integer values in [4, d]. However, the difference between PCLR and LAELR in this study is that instead of optimizing $\boldsymbol{W}$ only on the reconstruction loss (Equation 13), we optimize both $\boldsymbol{W}$ and $g(\cdot)$ jointly on the combined loss and corresponding objective, shown in Equations 14 and 15 respectively. This way, projection $\boldsymbol{W}$ is not purely optimized to explain the variance in $\boldsymbol{X}$, but also, for a part, to facilitate explanation of variance in $\boldsymbol{Y}$. If λ_{LAE} is very large the objective becomes similar to PCLR, whereas if λ_{LAE} is very small, the overall objective is similar to standard logistic regression. To empirically balance two loss functions we tune hyperparameter λ_{LAE} with a log-linear prior in the same range as the penalty of Lasso and Ridge: $[10^{-2}, 10^{3}]$.

$$\boldsymbol{W}^*, \boldsymbol{\gamma}^*, \gamma_0^* = \underset{\boldsymbol{W}, \boldsymbol{\gamma}, \gamma_0}{\arg\min}\, L_{\text{LAELR}}(\boldsymbol{W}, \boldsymbol{V}, \boldsymbol{\gamma}, \gamma_0) \tag{15}$$

Dropout regularization

Dropout training was proposed as a regularization method to prevent co-adaptation of weights in neural networks [6]. Dropout works in iterative gradient-based training procedures, like the one used in the current work (described in Appendix). When using dropout, a sub-model is randomly selected at each training iteration, effectively "dropping out" a random percentage δ of the model's coefficients. This selected sub-model is used to make predictions as part of that training iteration, and the involved coefficients are updated accordingly. Because at each iteration not all coefficients are involved in the model update, co-adaptation of the coefficients is disrupted. When training is completed, the coefficients are scaled down by a factor $1 - \delta$ to maintain the same expected output of the model during testing as during training (correcting for the fact that during training the full model was never used as a whole). The current work uses the dropout implementation in `PyTorch`, which is *inverted* dropout (used in most software implementations). In inverted dropout the coefficients are temporarily scaled during training by a factor $\frac{1}{1-\delta}$ instead of after training is completed. This way, no scaling is required when applying the model. For logistic regression[1], the loss function of dropout can be given by Equation 16, in which we abbreviate $f(\boldsymbol{x}_i, \boldsymbol{\beta}, \beta_0)$ as f_i for clarity [7]. The corresponding objective is given by Equation 17.

$$L_{\text{dropout}}(\boldsymbol{\beta}, \beta_0) = L_{\text{ML}}(\boldsymbol{\beta}, \beta_0) + \frac{1}{2}\frac{\delta}{1-\delta}\sum_{i=1}^{n}\sum_{j=1}^{d} f_i(1 - f_i)x_{ij}^2\beta_j^2 \tag{16}$$

$$\boldsymbol{W}^* = \underset{\boldsymbol{W}}{\arg\min}\, L_{\text{dropout}}(\boldsymbol{W}) \tag{17}$$

The loss function of dropout for logistic regression models can be summarized in two parts. First, like Ridge, it includes a quadratic penalty on the size of the coefficients, shown on the far right of the equation: β_j^2. Second, it includes an additional

[1]For other link functions than the logit function, the loss function of dropout is different. The more general formulation is provided in the original article by [6].

penalty discouraging moderate predictions during training (close to 0.5), shown by $f_i(1 - f_i)$. The degree of dropout regularization is determined by hyperparameter δ, which we tune using a linear prior over the interval [0.1, 0.5].

Non-negative logistic regression (LR_{NN})

Sometimes, the coefficient search space can be constrained based on prior knowledge, preventing the model's coefficient estimation procedure from exploring coefficients that are assumed invalid by the modeler. This can help reduce co-adaptation of coefficients, and their inflation, and may improve the model's predictive performance, if the assumption is valid.

In this study we explore the use of non-negativity constraints on dosage coefficients $\boldsymbol{\beta}_{\text{OAR}} \subseteq \boldsymbol{\beta}$, as we believe increasing dose to OAR should not result in a decrease in predicted risk of complications. The only difference in this method compared to standard logistic regression is that the feasible coefficient values for all dosage parameters are constrained to the non-negative region during loss minimization, shown in the objective in Eq. 18.

$$\boldsymbol{\beta}^*, \beta_0^* = \underset{\boldsymbol{\beta}, \beta_0}{\arg\min}\, L_{\text{ML}}(\boldsymbol{\beta}, \beta_0), \text{ with } \forall_{\beta_i \in \boldsymbol{\beta}_{\text{OAR}}} \beta_i \geq 0 \tag{18}$$

In terms of implementation, we enforce the constraint during our gradient-based minimization through gradient projection [8]: setting all negative dosage coefficients to 0 after each coefficient update.

References

1. Kingma DP, Ba J. Adam: A method for stochastic optimization. arXiv preprint arXiv:14126980. 2014;.
2. Morgan N, Bourlard H. Generalization and parameter estimation in feedforward nets: Some experiments. Advances in neural information processing systems. 1989;2:630–637.
3. Snoek J, Larochelle H, Adams RP. Practical Bayesian optimization of machine learning algorithms. Advances in Neural Information Processing Systems. 2012;p. 2951–2959.
4. Udell M. Generalized Low Rank Models. Stanford University; 2015.
5. Kunin D, Bloom J, Goeva A, Seed C. Loss Landscapes of Regularized Linear Autoencoders. International Conference on Machine Learning. 2019;p. 3560–3569.
6. Hinton GE, Srivastava N, Krizhevsky A, Sutskever I, Salakhutdinov RR. Improving neural networks by preventing co-adaptation of feature detectors. arXiv preprint arXiv:12070580. 2012;.
7. Wager S, Wang S, Liang PS. Dropout training as adaptive regularization. Advances in neural information processing systems. 2013;26:351–359.
8. Calamai PH, Moré JJ. Projected gradient methods for linearly constrained problems. Mathematical Programming. 1987;39(1):93–116.
