# Supplementary material for: Performance of binary prediction models in high-correlation low-dimensional settings: a comparison of methods
Source: Diagn Progn Res. 2022 Jan 11;6:1. doi: 10.1186/s41512-021-00115-5 (PMC8751246; doi:10.1186/s41512-021-00115-5)
Supplement: Supplementary file 4 — Additional file 4. Correlation plots per predictor set. [file 41512_2021_115_MOESM4_ESM.pdf]

Additional file 4

Correlation plots per predictor set.

In all correlation plots, negative correlations are indicated in blue, and positive correlations in red.

	AGE	Subm.L.Dm	Subm.R.Dm	Parotid.L.Dm	Parotid.R.Dm	XER.BSL.1
Subm.L.Dm	-0.28					
Subm.R.Dm	-0.29	0.89				
Parotid.L.Dm	-0.31	0.86	0.77			
Parotid.R.Dm	-0.30	0.72	0.83	0.78		
XER.BSL.1	0.04	0.15	0.10	0.08	0.00	
XER.BSL.2	0.02	-0.07	-0.08	-0.06	-0.07	-0.03

	AGE	Subm.L.Dm	Subm.R.Dm	Parotid.L.Dm	Parotid.R.Dm	PCM.Sup.Dm	PCM.Med.Dm	PCM.Inf.Dm	Supraglottic.Dm	OralCavity.Ext.Dm	GlotticArea.Dm	DYSFAGIE.BSL.1
Subm.L.Dm	-0.25											
Subm.R.Dm	-0.27	0.88										
Parotid.L.Dm	-0.26	0.85	0.72									
Parotid.R.Dm	-0.28	0.69	0.83	0.74								
PCM.Sup.Dm	-0.28	0.85	0.86	0.82	0.81							
PCM.Med.Dm	-0.25	0.87	0.89	0.74	0.72	0.79						
PCM.Inf.Dm	0.11	-0.11	-0.13	-0.11	-0.12	-0.35	0.11					
Supraglottic.Dm	-0.08	0.36	0.38	0.27	0.27	0.12	0.60	0.69				
OralCavity.Ext.Dm	-0.28	0.83	0.85	0.80	0.80	0.95	0.73	-0.40	0.07			
GlotticArea.Dm	0.15	-0.33	-0.34	-0.31	-0.31	-0.56	-0.16	0.89	0.54	-0.60		
DYSFAGIE.BSL.1	-0.04	0.24	0.25	0.25	0.21	0.24	0.23	-0.06	0.03	0.28	-0.12	
DYSFAGIE.BSL.2	-0.09	0.18	0.17	0.23	0.23	0.17	0.20	0.06	0.13	0.21	-0.01	-0.10

Figure 1 Correlation matrix for the A setting (left), and the C setting (right).

	AGE	Subm.L.Dm	Subm.L.V10	Subm.L.V30	Subm.L.V50	Subm.R.Dm	Subm.R.V10	Subm.R.V30	Subm.R.V50	Parotid.L.Dm	Parotid.L.V10	Parotid.L.V30	Parotid.L.V50	Parotid.R.Dm	Parotid.R.V10	Parotid.R.V30	Parotid.R.V50	XER.BSL.1
Subm.L.Dm	-0.28																	
Subm.L.V10	-0.25	0.94																
Subm.L.V30	-0.24	0.95	0.97															
Subm.L.V50	-0.28	0.94	0.82	0.85														
Subm.R.Dm	-0.29	0.89	0.90	0.89	0.83													
Subm.R.V10	-0.24	0.90	0.96	0.94	0.78	0.94												
Subm.R.V30	-0.24	0.90	0.96	0.94	0.79	0.95	0.99											
Subm.R.V50	-0.28	0.82	0.77	0.76	0.83	0.93	0.80	0.82										
Parotid.L.Dm	-0.31	0.86	0.76	0.77	0.83	0.77	0.74	0.74	0.74									
Parotid.L.V10	-0.29	0.89	0.84	0.84	0.84	0.82	0.81	0.81	0.77	0.93								
Parotid.L.V30	-0.30	0.81	0.71	0.73	0.79	0.71	0.68	0.69	0.70	0.98	0.89							
Parotid.L.V50	-0.29	0.67	0.52	0.54	0.69	0.55	0.50	0.50	0.58	0.91	0.72	0.90						
Parotid.R.Dm	-0.30	0.72	0.70	0.69	0.69	0.83	0.73	0.74	0.80	0.78	0.80	0.77	0.62					
Parotid.R.V10	-0.28	0.79	0.79	0.77	0.73	0.87	0.81	0.82	0.82	0.82	0.89	0.78	0.60	0.92				
Parotid.R.V30	-0.28	0.67	0.65	0.64	0.64	0.78	0.67	0.69	0.75	0.75	0.75	0.77	0.61	0.98	0.87			
Parotid.R.V50	-0.28	0.48	0.43	0.43	0.50	0.62	0.46	0.47	0.63	0.58	0.54	0.60	0.52	0.90	0.67	0.89		
XER.BSL.1	0.04	0.15	0.13	0.14	0.17	0.10	0.13	0.12	0.09	0.08	0.10	0.05	0.05	0.00	0.03	-0.02	-0.04	
XER.BSL.2	0.02	-0.07	-0.08	-0.07	-0.05	-0.08	-0.09	-0.08	-0.05	-0.06	-0.07	-0.06	-0.04	-0.07	-0.08	-0.06	-0.05	-0.03

Figure 2 Correlation matrix for the $B_{\triangle}$ setting.

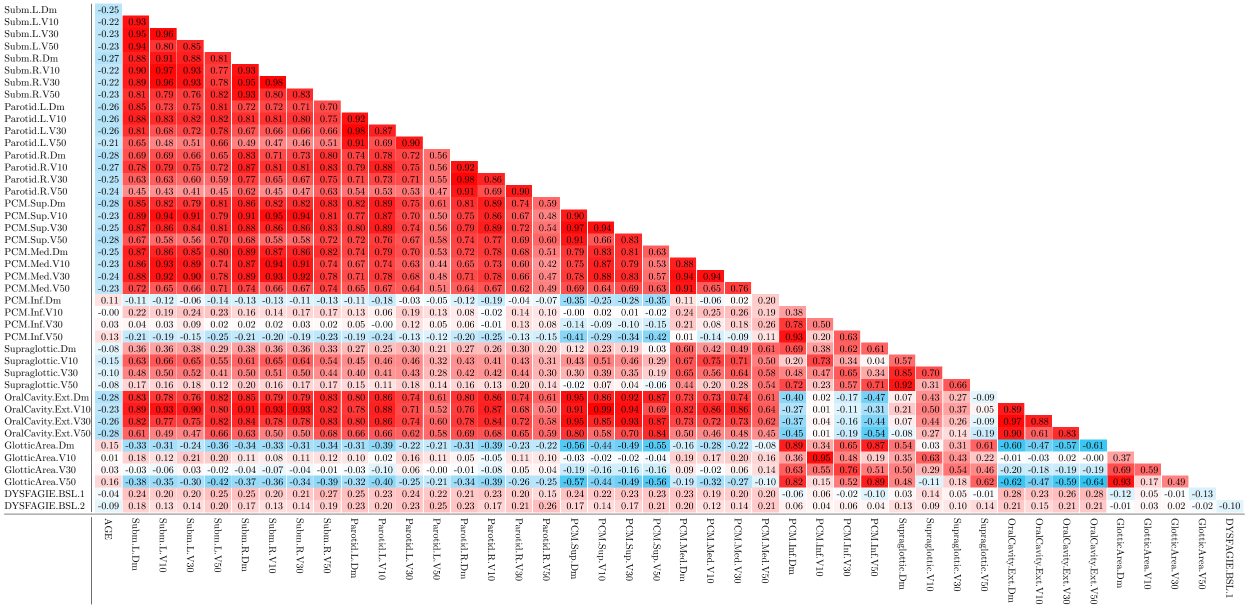

Figure 3 Correlation matrix for the $D_{\triangle}$ setting.
