# Supplementary material for: Performance of binary prediction models in high-correlation low-dimensional settings: a comparison of methods
Source: Diagn Progn Res. 2022 Jan 11;6:1. doi: 10.1186/s41512-021-00115-5 (PMC8751246; doi:10.1186/s41512-021-00115-5)
Supplement: Supplementary file 5 — Additional file 5. Obtained model coefficients per experimental setting, including the real data settings. [file 41512_2021_115_MOESM5_ESM.pdf]

Additional file 5

Obtained model coefficients per experimental setting, including the real data settings.

In this section, we report the mean coefficients of the estimated models in all simulated, and real-data settings, together with some general statistics about the coefficients: the sum, and proportion of all negative dose-coefficients ($\sum_{\beta_{<-0.01}}$ and $P_{\beta_{<-0.01}}$ respectively), and the sum and proportion of all positive dose-coefficients ($\sum_{\beta_{>0.01}}$ and $P_{\beta_{>0.01}}$ respectively).

Table 1 **Mean model coefficients for A.**

	LR		Lasso		Ridge		ElasticNet		PCLR		LAELR		Dropout		LRnn	
$\sum_{\beta_{<-0.01}}$	-0.13	(−0.71,0.00)	-0.01	(0.00,0.00)	-0.01	(−0.09,0.00)	0.00	(0.00,0.00)	-0.04	(−0.66,0.00)	-0.03	(−0.42,0.00)	-0.00	(0.00,0.00)	0.00	(0.00,0.00)
$\sum_{\beta_{>0.01}}$	1.48	(1.10,2.04)	1.24	(0.94,1.52)	1.20	(0.93,1.56)	1.20	(0.94,1.50)	1.38	(1.06,2.04)	1.37	(1.06,1.94)	1.20	(0.83,1.55)	1.36	(1.09,1.63)
$P_{\beta_{<-0.01}}$	0.16	(0.00,0.50)	0.01	(0.00,0.00)	0.01	(0.00,0.25)	0.00	(0.00,0.00)	0.04	(0.00,0.50)	0.03	(0.00,0.25)	0.00	(0.00,0.00)	0.00	(0.00,0.00)
$P_{\beta_{>0.01}}$	0.83	(0.50,1.00)	0.86	(0.50,1.00)	0.99	(0.75,1.00)	0.96	(0.75,1.00)	0.96	(0.50,1.00)	0.96	(0.75,1.00)	1.00	(1.00,1.00)	0.84	(0.50,1.00)
Intercept	-1.32	(−1.58,−1.12)	-1.27	(−1.53,−1.07)	-1.26	(−1.52,−1.08)	-1.26	(−1.50,−1.09)	-1.32	(−1.58,−1.12)	-1.32	(−1.57,−1.12)	-1.27	(−1.52,−1.06)	-1.32	(−1.58,−1.12)
AGE	-0.05	(−0.25,0.15)	-0.04	(−0.23,0.10)	-0.05	(−0.22,0.11)	-0.04	(−0.21,0.10)	-0.05	(−0.26,0.14)	-0.05	(−0.26,0.15)	-0.06	(−0.23,0.12)	-0.05	(−0.25,0.14)
Subm.L.Dm	0.19	(−0.51,0.82)	0.21	(−0.00,0.72)	0.24	(−0.00,0.39)	0.22	(0.00,0.55)	0.28	(−0.36,0.63)	0.28	(−0.36,0.44)	0.26	(0.09,0.40)	0.23	(0.00,0.81)
Subm.R.Dm	0.38	(−0.35,1.00)	0.34	(−0.00,0.86)	0.31	(0.05,0.68)	0.32	(0.01,0.81)	0.38	(−0.00,0.85)	0.37	(−0.08,0.85)	0.30	(0.10,0.58)	0.36	(0.00,0.94)
Parotid.L.Dm	0.39	(−0.09,0.95)	0.33	(0.00,0.77)	0.31	(0.08,0.67)	0.32	(0.02,0.70)	0.34	(0.01,0.82)	0.33	(0.00,0.80)	0.31	(0.08,0.56)	0.37	(0.00,0.78)
Parotid.R.Dm	0.40	(−0.18,0.91)	0.35	(−0.00,0.78)	0.33	(0.08,0.58)	0.34	(0.01,0.63)	0.35	(−0.08,0.74)	0.36	(0.10,0.74)	0.33	(0.06,0.67)	0.40	(0.00,0.81)
XER.BSL.2	0.25	(0.02,0.47)	0.18	(−0.01,0.43)	0.19	(0.01,0.39)	0.19	(0.01,0.39)	0.24	(0.02,0.46)	0.24	(0.02,0.45)	0.18	(0.02,0.35)	0.25	(0.02,0.47)
XER.BSL.3	0.13	(−0.04,0.30)	0.08	(−0.01,0.26)	0.09	(−0.03,0.26)	0.08	(−0.03,0.24)	0.12	(−0.04,0.29)	0.12	(−0.05,0.30)	0.09	(−0.05,0.27)	0.13	(−0.04,0.30)

Table 2 **Mean model coefficients for $A_{\triangle}$.**

	LR		Lasso		Ridge		ElasticNet		PCLR		LAELR		Dropout		LRnn	
$\sum_{\beta_{<-0.01}}$	-0.65	(−3.03,0.00)	0.00	(0.00,0.00)	-0.01	(−0.15,0.00)	0.00	(0.00,0.00)	-0.30	(−3.76,0.00)	-0.34	(−3.66,0.00)	0.00	(0.00,0.00)	0.00	(0.00,0.00)
$\sum_{\beta_{>0.01}}$	2.08	(1.17,3.74)	1.27	(0.97,1.65)	1.27	(1.00,1.64)	1.26	(1.00,1.63)	1.74	(1.15,5.87)	1.78	(1.15,5.81)	1.28	(0.99,1.68)	1.43	(1.13,1.84)
$P_{\beta_{<-0.01}}$	0.28	(0.00,0.50)	0.00	(0.00,0.00)	0.01	(0.00,0.25)	0.00	(0.00,0.00)	0.06	(0.00,0.50)	0.08	(0.00,0.50)	0.00	(0.00,0.00)	0.00	(0.00,0.00)
$P_{\beta_{>0.01}}$	0.71	(0.50,1.00)	0.85	(0.50,1.00)	0.99	(0.75,1.00)	0.97	(0.75,1.00)	0.93	(0.50,1.00)	0.92	(0.50,1.00)	1.00	(1.00,1.00)	0.70	(0.50,1.00)
Intercept	-1.35	(−1.56,−1.04)	-1.28	(−1.49,−1.02)	-1.28	(−1.48,−1.01)	-1.28	(−1.48,−1.01)	-1.34	(−1.56,−1.04)	-1.34	(−1.56,−1.04)	-1.29	(−1.53,−1.01)	-1.34	(−1.56,−1.04)
AGE	0.02	(−0.77,0.85)	-0.01	(−0.16,0.09)	-0.01	(−0.20,0.14)	-0.01	(−0.18,0.14)	0.04	(−0.67,1.22)	0.04	(−0.73,1.19)	-0.00	(−0.20,0.17)	0.02	(−0.24,0.35)
Subm.L.Dm	0.26	(−2.04,2.82)	0.15	(−0.00,0.73)	0.24	(0.13,0.37)	0.20	(0.00,0.38)	0.32	(−2.22,3.98)	0.31	(−2.09,3.95)	0.26	(0.12,0.39)	0.25	(0.00,0.97)
Subm.R.Dm	0.26	(−1.57,1.86)	0.35	(−0.00,1.01)	0.31	(0.15,0.59)	0.33	(0.00,0.87)	0.28	(−3.00,1.89)	0.29	(−2.49,1.77)	0.32	(0.12,0.52)	0.29	(0.00,1.11)
Parotid.L.Dm	0.41	(−0.65,1.40)	0.38	(0.00,1.00)	0.33	(0.09,0.59)	0.35	(0.00,0.88)	0.37	(−0.83,1.52)	0.39	(−0.75,1.49)	0.33	(0.11,0.59)	0.40	(0.00,1.12)
Parotid.R.Dm	0.51	(−0.18,1.28)	0.39	(0.01,0.83)	0.38	(0.22,0.67)	0.38	(0.15,0.69)	0.47	(0.10,1.50)	0.46	(0.02,1.39)	0.37	(0.21,0.78)	0.49	(0.07,0.97)
XER.BSL.2	0.14	(−0.08,0.44)	0.09	(−0.03,0.38)	0.12	(−0.06,0.35)	0.10	(−0.02,0.38)	0.13	(−0.08,0.42)	0.13	(−0.09,0.42)	0.11	(−0.05,0.35)	0.14	(−0.08,0.43)
XER.BSL.3	0.06	(−0.12,0.26)	0.03	(−0.08,0.18)	0.03	(−0.10,0.19)	0.03	(−0.09,0.19)	0.05	(−0.12,0.26)	0.05	(−0.14,0.26)	0.04	(−0.12,0.19)	0.06	(−0.12,0.27)

Table 3 **Mean model coefficients for B.**

	LR		Lasso		Ridge		ElasticNet		PCLR		LAELR		Dropout		LRnn	
$\sum_{\beta_{<-0.01}}$	-1.18	(−2.25,−0.34)	-0.01	(−0.20,0.00)	-0.04	(−0.22,0.00)	-0.02	(−0.17,0.00)	-0.06	(−0.56,0.00)	-0.07	(−0.46,0.00)	-0.05	(−0.22,0.00)	0.00	(0.00,0.00)
$\sum_{\beta_{>0.01}}$	2.81	(1.98,3.92)	1.39	(1.00,1.82)	1.44	(1.01,1.81)	1.42	(1.04,1.78)	1.65	(1.26,2.51)	1.65	(1.21,2.25)	1.51	(1.16,1.98)	1.65	(1.26,2.02)
$P_{\beta_{<-0.01}}$	0.34	(0.19,0.50)	0.01	(0.00,0.06)	0.05	(0.00,0.19)	0.03	(0.00,0.19)	0.04	(0.00,0.25)	0.05	(0.00,0.25)	0.06	(0.00,0.19)	0.00	(0.00,0.00)
$P_{\beta_{>0.01}}$	0.63	(0.44,0.81)	0.65	(0.44,0.88)	0.92	(0.75,1.00)	0.83	(0.44,1.00)	0.94	(0.69,1.00)	0.93	(0.62,1.00)	0.89	(0.69,1.00)	0.56	(0.44,0.75)
Intercept	-1.37	(−1.71,−1.10)	-1.27	(−1.55,−1.02)	-1.25	(−1.53,−1.01)	-1.26	(−1.53,−1.03)	-1.33	(−1.64,−1.08)	-1.33	(−1.64,−1.08)	-1.29	(−1.61,−1.04)	-1.35	(−1.67,−1.09)
AGE	-0.03	(−0.31,0.28)	-0.02	(−0.20,0.17)	-0.03	(−0.18,0.13)	-0.03	(−0.17,0.12)	-0.04	(−0.23,0.20)	-0.03	(−0.27,0.21)	-0.03	(−0.19,0.12)	-0.02	(−0.25,0.27)
Subm.L.Dm	0.05	(−1.12,1.05)	0.08	(−0.00,0.47)	0.08	(−0.02,0.20)	0.09	(−0.02,0.24)	0.10	(0.01,0.18)	0.10	(−0.02,0.24)	0.09	(−0.02,0.24)	0.06	(0.00,0.53)
Subm.L.V10	0.12	(−0.29,0.49)	0.07	(−0.01,0.30)	0.07	(−0.04,0.21)	0.07	(−0.02,0.24)	0.07	(−0.11,0.24)	0.07	(−0.10,0.25)	0.08	(−0.04,0.26)	0.10	(0.00,0.40)
Subm.L.V30	0.04	(−0.46,0.47)	0.05	(−0.01,0.31)	0.06	(−0.04,0.20)	0.06	(−0.03,0.21)	0.07	(−0.09,0.21)	0.07	(−0.07,0.20)	0.06	(−0.08,0.18)	0.06	(0.00,0.35)
Subm.L.V50	0.10	(−0.23,0.47)	0.07	(−0.01,0.27)	0.08	(−0.02,0.17)	0.07	(−0.03,0.21)	0.09	(−0.09,0.26)	0.09	(−0.10,0.23)	0.08	(−0.04,0.19)	0.09	(0.00,0.33)
Subm.R.Dm	0.16	(−0.47,0.81)	0.10	(−0.00,0.44)	0.10	(0.01,0.25)	0.10	(0.00,0.27)	0.12	(−0.02,0.27)	0.12	(−0.01,0.27)	0.12	(0.00,0.32)	0.12	(0.00,0.53)
Subm.R.V10	0.05	(−0.45,0.51)	0.07	(−0.01,0.35)	0.07	(−0.04,0.18)	0.07	(−0.04,0.21)	0.09	(0.01,0.26)	0.09	(−0.00,0.21)	0.08	(−0.05,0.20)	0.08	(0.00,0.41)
Subm.R.V30	0.07	(−0.51,0.56)	0.08	(−0.01,0.41)	0.08	(−0.10,0.18)	0.08	(−0.03,0.26)	0.09	(0.02,0.17)	0.09	(−0.09,0.19)	0.08	(−0.05,0.21)	0.08	(0.00,0.38)
Subm.R.V50	0.12	(−0.29,0.44)	0.10	(−0.00,0.33)	0.10	(−0.01,0.22)	0.10	(−0.00,0.24)	0.11	(0.02,0.24)	0.12	(−0.03,0.24)	0.10	(−0.05,0.25)	0.12	(0.00,0.37)
Parotid.L.Dm	0.09	(−0.54,0.74)	0.07	(−0.00,0.26)	0.08	(−0.06,0.16)	0.08	(−0.01,0.17)	0.08	(−0.02,0.18)	0.10	(−0.03,0.20)	0.09	(−0.04,0.18)	0.09	(0.00,0.34)
Parotid.L.V10	0.14	(−0.28,0.57)	0.11	(−0.01,0.37)	0.10	(−0.04,0.23)	0.10	(−0.05,0.27)	0.10	(−0.16,0.36)	0.10	(−0.09,0.34)	0.11	(−0.02,0.25)	0.14	(0.00,0.43)
Parotid.L.V30	0.17	(−0.43,0.73)	0.11	(−0.00,0.37)	0.10	(−0.02,0.23)	0.10	(−0.00,0.25)	0.10	(0.02,0.22)	0.10	(−0.00,0.22)	0.10	(−0.03,0.27)	0.12	(0.00,0.40)
Parotid.L.V50	0.06	(−0.55,0.60)	0.05	(−0.03,0.29)	0.06	(−0.11,0.18)	0.05	(−0.08,0.23)	0.07	(−0.08,0.21)	0.06	(−0.21,0.23)	0.06	(−0.09,0.19)	0.07	(0.00,0.35)
Parotid.R.Dm	0.15	(−0.68,0.95)	0.08	(−0.00,0.37)	0.10	(0.00,0.21)	0.09	(−0.00,0.27)	0.12	(0.01,0.22)	0.13	(0.02,0.26)	0.10	(−0.00,0.23)	0.10	(0.00,0.40)
Parotid.R.V10	0.19	(−0.27,0.68)	0.17	(−0.00,0.52)	0.13	(0.04,0.27)	0.15	(0.01,0.38)	0.13	(0.05,0.37)	0.12	(0.02,0.34)	0.14	(0.00,0.32)	0.19	(0.00,0.54)
Parotid.R.V30	0.09	(−0.55,0.81)	0.13	(−0.00,0.47)	0.11	(−0.00,0.23)	0.11	(−0.00,0.35)	0.13	(0.05,0.26)	0.12	(−0.01,0.26)	0.11	(−0.01,0.24)	0.12	(0.00,0.51)
Parotid.R.V50	0.06	(−0.75,0.71)	0.08	(−0.01,0.36)	0.08	(−0.04,0.20)	0.08	(−0.02,0.30)	0.11	(−0.04,0.22)	0.10	(−0.06,0.22)	0.08	(−0.05,0.21)	0.11	(0.00,0.43)
XER.BSL.2	0.43	(0.18,0.70)	0.31	(0.08,0.56)	0.24	(0.09,0.51)	0.26	(0.11,0.52)	0.38	(0.00,0.67)	0.36	(0.00,0.68)	0.23	(0.07,0.46)	0.42	(0.18,0.69)
XER.BSL.3	0.16	(−0.03,0.41)	0.09	(−0.01,0.31)	0.09	(−0.01,0.24)	0.09	(−0.01,0.25)	0.15	(−0.06,0.38)	0.14	(−0.09,0.37)	0.08	(−0.01,0.22)	0.16	(−0.03,0.39)

Table 4 Mean model coefficients for $B_{\triangle}$.

	LR		Lasso		Ridge		ElasticNet		PCLR		LAELR		Dropout		LRnn	
$\sum_{\beta_{<-0.01}}$	-3.99	(−7.41,−1.42)	-0.10	(−1.37,0.00)	-0.09	(−0.93,0.00)	-0.03	(−0.50,0.00)	-0.14	(−1.33,0.00)	-0.30	(−4.09,0.00)	-0.06	(−0.58,0.00)	0.00	(0.00,0.00)
$\sum_{\beta_{>0.01}}$	5.52	(2.92,8.67)	1.43	(0.97,2.89)	1.46	(1.04,2.51)	1.39	(1.01,1.96)	1.68	(1.26,3.17)	1.83	(1.23,5.58)	1.50	(1.09,2.09)	1.57	(1.23,2.00)
$P_{\beta_{<-0.01}}$	0.45	(0.31,0.62)	0.02	(0.00,0.25)	0.04	(0.00,0.38)	0.01	(0.00,0.19)	0.06	(0.00,0.44)	0.06	(0.00,0.50)	0.06	(0.00,0.31)	0.00	(0.00,0.00)
$P_{\beta_{>0.01}}$	0.54	(0.38,0.69)	0.60	(0.31,0.88)	0.95	(0.56,1.00)	0.87	(0.31,1.00)	0.93	(0.50,1.00)	0.93	(0.50,1.00)	0.90	(0.69,1.00)	0.39	(0.25,0.56)
Intercept	-1.39	(−1.66,−1.09)	-1.29	(−1.52,−1.02)	-1.29	(−1.53,−1.01)	-1.29	(−1.55,−1.01)	-1.35	(−1.61,−1.06)	-1.35	(−1.61,−1.06)	-1.32	(−1.57,−1.04)	-1.37	(−1.63,−1.07)
AGE	-0.04	(−0.29,0.15)	-0.03	(−0.21,0.06)	-0.03	(−0.21,0.07)	-0.03	(−0.20,0.08)	-0.02	(−0.25,0.16)	-0.02	(−0.27,0.10)	-0.03	(−0.20,0.11)	-0.04	(−0.26,0.17)
Subm.L.Dm	0.06	(−1.60,1.40)	0.08	(−0.00,0.62)	0.08	(−0.03,0.19)	0.08	(−0.00,0.25)	0.10	(−0.08,0.26)	0.09	(−0.14,0.26)	0.08	(−0.08,0.20)	0.07	(0.00,0.51)
Subm.L.V10	0.00	(−1.51,1.65)	0.06	(−0.01,0.46)	0.07	(−0.04,0.14)	0.07	(−0.01,0.34)	0.09	(−0.03,0.17)	0.09	(−0.13,0.18)	0.07	(−0.05,0.18)	0.07	(0.00,0.56)
Subm.L.V30	-0.00	(−1.09,1.02)	0.04	(−0.01,0.38)	0.05	(−0.24,0.14)	0.06	(−0.01,0.16)	0.07	(−0.12,0.15)	0.06	(−0.76,0.23)	0.07	(−0.09,0.18)	0.05	(0.00,0.37)
Subm.L.V50	0.07	(−0.88,1.01)	0.06	(−0.07,0.37)	0.07	(−0.08,0.16)	0.06	(−0.07,0.18)	0.07	(−0.35,0.28)	0.09	(−0.25,0.38)	0.08	(−0.07,0.23)	0.08	(0.00,0.41)
Subm.R.Dm	0.24	(−1.33,2.12)	0.14	(−0.00,0.89)	0.12	(0.03,0.43)	0.11	(0.01,0.47)	0.12	(0.04,0.18)	0.15	(0.03,0.90)	0.11	(0.02,0.23)	0.10	(0.00,0.72)
Subm.R.V10	0.00	(−1.17,1.40)	0.04	(−0.00,0.51)	0.08	(−0.01,0.20)	0.07	(−0.00,0.19)	0.10	(−0.04,0.22)	0.10	(−0.17,0.44)	0.09	(−0.04,0.20)	0.08	(0.00,0.58)
Subm.R.V30	0.22	(−1.19,1.60)	0.11	(−0.00,0.64)	0.09	(0.02,0.27)	0.09	(0.00,0.35)	0.09	(−0.13,0.20)	0.09	(−0.47,0.33)	0.09	(−0.04,0.25)	0.15	(0.00,0.73)
Subm.R.V50	0.07	(−1.10,0.99)	0.11	(−0.00,0.53)	0.09	(−0.15,0.19)	0.10	(−0.00,0.29)	0.09	(−0.19,0.27)	0.07	(−0.43,0.17)	0.10	(−0.06,0.24)	0.13	(0.00,0.57)
Parotid.L.Dm	0.35	(−2.12,2.97)	0.09	(−0.00,0.50)	0.10	(0.04,0.34)	0.09	(0.00,0.21)	0.11	(0.01,0.29)	0.13	(0.03,0.56)	0.10	(−0.01,0.20)	0.05	(0.00,0.38)
Parotid.L.V10	0.11	(−1.00,0.91)	0.15	(−0.00,0.79)	0.12	(0.02,0.64)	0.13	(0.00,0.73)	0.13	(0.05,0.57)	0.14	(0.05,0.80)	0.12	(−0.02,0.34)	0.16	(0.00,0.73)
Parotid.L.V30	0.08	(−1.20,1.82)	0.11	(−0.00,0.57)	0.09	(0.01,0.32)	0.10	(0.00,0.53)	0.09	(−0.15,0.30)	0.09	(−0.17,0.47)	0.10	(−0.02,0.34)	0.12	(0.00,0.57)
Parotid.L.V50	-0.06	(−1.31,0.93)	0.03	(−0.01,0.31)	0.05	(−0.16,0.14)	0.05	(−0.14,0.16)	0.07	(−0.25,0.25)	0.06	(−0.37,0.21)	0.05	(−0.10,0.18)	0.09	(0.00,0.36)
Parotid.R.Dm	0.08	(−3.11,3.09)	0.11	(−0.01,0.42)	0.09	(0.00,0.14)	0.09	(0.00,0.18)	0.11	(0.01,0.18)	0.09	(−0.27,0.21)	0.10	(0.00,0.19)	0.04	(0.00,0.33)
Parotid.R.V10	0.16	(−0.99,1.40)	0.14	(−0.01,0.49)	0.11	(0.02,0.29)	0.12	(0.00,0.36)	0.13	(−0.01,0.52)	0.11	(−0.08,0.54)	0.13	(−0.01,0.38)	0.17	(0.00,0.57)
Parotid.R.V30	0.07	(−1.48,1.93)	0.07	(−0.01,0.46)	0.09	(−0.03,0.17)	0.09	(−0.00,0.29)	0.10	(−0.32,0.25)	0.09	(−0.36,0.43)	0.09	(−0.03,0.20)	0.13	(0.00,0.51)
Parotid.R.V50	0.08	(−1.25,1.62)	0.04	(−0.01,0.32)	0.07	(−0.08,0.20)	0.06	(−0.04,0.21)	0.08	(−0.22,0.30)	0.09	(−0.19,0.40)	0.06	(−0.07,0.17)	0.10	(0.00,0.39)
XER.BSL.2	0.20	(−0.01,0.40)	0.12	(−0.00,0.35)	0.11	(−0.01,0.34)	0.12	(−0.00,0.29)	0.14	(−0.04,0.37)	0.12	(−0.08,0.37)	0.11	(−0.03,0.30)	0.20	(−0.03,0.37)
XER.BSL.3	0.09	(−0.15,0.33)	0.04	(−0.05,0.22)	0.05	(−0.05,0.21)	0.05	(−0.05,0.24)	0.06	(−0.13,0.29)	0.05	(−0.14,0.27)	0.05	(−0.07,0.21)	0.09	(−0.12,0.32)

Table 5 Mean model coefficients for C.

	LR		Lasso		Ridge		ElasticNet		PCLR		LAELR		Dropout		LRnn	
$\sum_{\beta_{<-0.01}}$	-1.02	(−2.38,−0.21)	-0.05	(−0.50,0.00)	-0.08	(−0.55,0.00)	-0.06	(−0.54,0.00)	-0.18	(−1.69,0.00)	-0.15	(−1.65,0.00)	-0.06	(−0.25,0.00)	0.00	(0.00,0.00)
$\sum_{\beta_{>0.01}}$	3.48	(2.44,5.28)	2.07	(1.50,3.11)	2.06	(1.58,3.00)	2.06	(1.49,2.92)	2.49	(1.83,4.17)	2.46	(1.81,4.16)	2.01	(1.50,2.73)	2.52	(1.93,3.62)
$P_{\beta_{<-0.01}}$	0.32	(0.20,0.50)	0.02	(0.00,0.20)	0.08	(0.00,0.30)	0.05	(0.00,0.20)	0.08	(0.00,0.30)	0.07	(0.00,0.30)	0.07	(0.00,0.20)	0.00	(0.00,0.00)
$P_{\beta_{>0.01}}$	0.67	(0.50,0.80)	0.69	(0.50,0.90)	0.90	(0.70,1.00)	0.85	(0.50,1.00)	0.91	(0.60,1.00)	0.91	(0.70,1.00)	0.92	(0.80,1.00)	0.64	(0.40,0.90)
Intercept	-2.96	(−3.58,−2.49)	-2.69	(−3.16,−2.28)	-2.66	(−3.12,−2.27)	-2.67	(−3.14,−2.28)	-2.89	(−3.48,−2.44)	-2.88	(−3.52,−2.44)	-2.69	(−3.19,−2.33)	-2.93	(−3.56,−2.47)
AGE	0.01	(−0.28,0.25)	0.00	(−0.17,0.18)	-0.01	(−0.19,0.17)	-0.01	(−0.18,0.18)	0.03	(−0.30,0.27)	0.04	(−0.31,0.27)	-0.01	(−0.22,0.17)	0.02	(−0.25,0.26)
Subm.L.Dm	0.22	(−0.75,1.07)	0.19	(−0.00,0.66)	0.22	(−0.12,0.53)	0.22	(−0.07,0.54)	0.28	(−0.17,0.88)	0.28	(−0.16,0.80)	0.23	(0.05,0.44)	0.21	(0.00,0.64)
Subm.R.Dm	0.26	(−0.82,1.37)	0.22	(−0.00,0.89)	0.24	(0.03,0.55)	0.24	(−0.00,0.74)	0.28	(−0.10,0.69)	0.27	(−0.50,0.71)	0.25	(0.05,0.57)	0.25	(0.00,1.01)
Parotid.L.Dm	0.12	(−0.54,0.86)	0.14	(−0.03,0.60)	0.18	(−0.07,0.43)	0.17	(−0.05,0.46)	0.21	(−0.33,0.58)	0.21	(−0.30,0.41)	0.19	(−0.10,0.40)	0.15	(0.00,0.63)
Parotid.R.Dm	0.21	(−0.52,1.05)	0.23	(−0.00,0.73)	0.23	(0.00,0.56)	0.22	(−0.00,0.64)	0.27	(−0.16,0.72)	0.28	(−0.13,0.71)	0.22	(−0.05,0.42)	0.22	(0.00,0.78)
PCM.Sup.Dm	0.27	(−0.95,1.56)	0.20	(−0.00,0.85)	0.24	(0.01,0.42)	0.23	(0.00,0.59)	0.25	(−0.02,0.80)	0.24	(−0.15,0.77)	0.24	(0.05,0.39)	0.33	(0.00,1.10)
PCM.Med.Dm	0.22	(−0.83,1.51)	0.29	(−0.00,1.00)	0.23	(−0.00,0.47)	0.25	(−0.00,0.72)	0.26	(−0.71,0.55)	0.29	(−0.38,0.55)	0.23	(0.00,0.56)	0.23	(0.00,1.04)
PCM.Inf.Dm	0.33	(−0.43,1.19)	0.13	(−0.01,0.50)	0.15	(−0.06,0.40)	0.15	(−0.03,0.42)	0.18	(−0.03,0.58)	0.15	(0.01,0.52)	0.13	(−0.04,0.45)	0.21	(0.00,0.63)
Supraglottic.Dm	0.19	(−0.78,1.02)	0.18	(−0.08,0.65)	0.17	(−0.17,0.47)	0.17	(−0.21,0.55)	0.21	(−0.60,0.65)	0.21	(−0.58,0.61)	0.18	(−0.20,0.44)	0.20	(0.00,0.74)
OralCavity.Ext.Dm	0.62	(−0.75,1.86)	0.40	(0.00,1.27)	0.30	(0.13,0.89)	0.32	(0.00,1.00)	0.33	(0.19,1.05)	0.33	(0.19,1.16)	0.27	(0.11,0.72)	0.57	(0.00,1.55)
GlotticArea.Dm	0.02	(−0.79,1.03)	0.03	(−0.37,0.50)	0.02	(−0.19,0.32)	0.03	(−0.20,0.39)	0.03	(−0.37,0.57)	0.04	(−0.19,0.56)	0.02	(−0.24,0.30)	0.15	(0.00,0.83)
DYSFAGIE..BSL.2	0.23	(−0.06,0.50)	0.15	(−0.01,0.42)	0.17	(−0.04,0.38)	0.17	(−0.01,0.37)	0.19	(−0.14,0.47)	0.16	(−0.16,0.48)	0.17	(−0.01,0.39)	0.23	(−0.08,0.49)
DYSFAGIE..BSL.3	0.42	(0.12,0.81)	0.31	(0.01,0.61)	0.30	(0.09,0.56)	0.30	(0.08,0.55)	0.38	(0.10,0.77)	0.36	(0.10,0.80)	0.29	(0.08,0.61)	0.42	(0.13,0.80)

Table 6 Mean model coefficients for $C_{\triangle}$.

	LR		Lasso		Ridge		ElasticNet		PCLR		LAELR		Dropout		LRnn	
$\sum_{\beta_{<-0.01}}$	-3.14	(−7.37,−0.74)	-0.08	(−1.10,0.00)	-0.11	(−1.31,0.00)	-0.06	(−0.61,0.00)	-0.31	(−3.40,0.00)	-0.39	(−4.87,0.00)	-0.08	(−0.50,0.00)	0.00	(0.00,0.00)
$\sum_{\beta_{>0.01}}$	5.40	(2.87,10.86)	1.88	(1.24,3.37)	1.88	(1.30,3.21)	1.82	(1.33,2.82)	2.36	(1.63,6.30)	2.44	(1.59,6.79)	1.84	(1.23,2.72)	2.33	(1.62,3.33)
$P_{\beta_{<-0.01}}$	0.40	(0.20,0.60)	0.03	(0.00,0.20)	0.08	(0.00,0.40)	0.04	(0.00,0.20)	0.10	(0.00,0.50)	0.10	(0.00,0.50)	0.09	(0.00,0.30)	0.00	(0.00,0.00)
$P_{\beta_{>0.01}}$	0.60	(0.40,0.80)	0.60	(0.30,0.90)	0.90	(0.60,1.00)	0.82	(0.40,1.00)	0.88	(0.50,1.00)	0.88	(0.50,1.00)	0.89	(0.70,1.00)	0.48	(0.30,0.70)
Intercept	-2.89	(−3.43,−2.41)	-2.65	(−3.22,−2.22)	-2.62	(−3.10,−2.24)	-2.62	(−3.06,−2.21)	-2.82	(−3.40,−2.37)	-2.82	(−3.39,−2.37)	-2.66	(−3.13,−2.26)	-2.86	(−3.41,−2.40)
AGE	0.07	(−0.70,0.79)	0.02	(−0.18,0.34)	0.02	(−0.22,0.29)	0.02	(−0.18,0.28)	0.07	(−0.25,0.36)	0.09	(−0.26,0.59)	0.02	(−0.20,0.25)	0.06	(−0.26,0.42)
Subm.L.Dm	0.22	(−2.42,3.20)	0.10	(−0.00,0.54)	0.15	(−0.15,0.38)	0.14	(−0.01,0.39)	0.16	(−0.39,0.44)	0.24	(−0.34,1.86)	0.14	(−0.17,0.33)	0.17	(0.00,0.97)
Subm.R.Dm	0.16	(−2.67,2.36)	0.12	(−0.00,0.88)	0.19	(−0.17,0.42)	0.19	(0.00,0.43)	0.19	(−0.49,0.36)	0.21	(−0.62,0.49)	0.20	(−0.01,0.43)	0.14	(0.00,0.98)
Parotid.L.Dm	0.00	(−1.63,1.55)	0.18	(−0.01,0.79)	0.18	(−0.03,0.36)	0.17	(−0.00,0.53)	0.19	(−0.43,0.62)	0.17	(−0.90,0.40)	0.18	(−0.04,0.42)	0.15	(0.00,0.79)
Parotid.R.Dm	0.23	(−1.26,1.62)	0.30	(−0.04,0.97)	0.24	(−0.10,0.52)	0.26	(−0.00,0.73)	0.26	(−0.32,0.78)	0.28	(−0.27,0.95)	0.25	(−0.05,0.61)	0.29	(0.00,1.01)
PCM.Sup.Dm	0.47	(−2.25,3.16)	0.21	(−0.00,0.96)	0.25	(0.13,0.59)	0.24	(0.01,0.69)	0.33	(0.11,1.48)	0.29	(0.02,1.23)	0.24	(0.11,0.43)	0.24	(0.00,1.20)
PCM.Med.Dm	0.12	(−2.38,2.67)	0.39	(−0.00,1.26)	0.24	(0.10,0.51)	0.26	(0.00,0.76)	0.31	(0.08,0.57)	0.25	(−0.68,0.53)	0.24	(0.08,0.47)	0.26	(0.00,1.27)
PCM.Inf.Dm	0.33	(−1.20,2.03)	0.08	(−0.01,0.80)	0.09	(−0.06,0.28)	0.07	(−0.13,0.36)	0.11	(−0.48,0.44)	0.09	(−0.52,0.29)	0.09	(−0.09,0.31)	0.18	(0.00,0.75)
Supraglottic.Dm	0.23	(−1.02,1.96)	0.20	(−0.00,0.81)	0.22	(0.04,0.74)	0.22	(0.00,0.80)	0.29	(−0.06,1.11)	0.32	(0.11,1.58)	0.23	(0.03,0.67)	0.20	(0.00,0.76)
OralCavity.Ext.Dm	0.55	(−2.06,3.07)	0.25	(−0.00,1.30)	0.23	(0.10,0.75)	0.22	(0.00,0.60)	0.26	(−0.10,0.99)	0.25	(−0.34,1.04)	0.22	(0.03,0.44)	0.57	(0.00,1.86)
GlotticArea.Dm	-0.06	(−1.19,1.08)	-0.02	(−0.58,0.33)	-0.02	(−0.30,0.16)	-0.02	(−0.33,0.15)	-0.04	(−0.71,0.25)	-0.04	(−0.77,0.16)	-0.03	(−0.30,0.14)	0.11	(0.00,0.49)
DYSFAGIE..BSL.2	0.15	(−0.16,0.49)	0.11	(−0.01,0.41)	0.14	(−0.02,0.33)	0.13	(−0.01,0.39)	0.13	(−0.11,0.46)	0.13	(−0.08,0.44)	0.15	(−0.09,0.40)	0.15	(−0.14,0.50)
DYSFAGIE..BSL.3	0.27	(−0.12,0.62)	0.20	(−0.02,0.46)	0.21	(−0.02,0.41)	0.21	(−0.01,0.45)	0.26	(−0.05,0.54)	0.26	(−0.04,0.55)	0.21	(−0.02,0.43)	0.26	(−0.12,0.55)

Table 7 Mean model coefficients for D.

	LR		Lasso		Ridge		ElasticNet		PCLR		LAELR		Dropout		LRnn	
$\sum_{\beta<-0.01}$	-7.10	(−10.95,−4.18)	-0.05	(−0.29,0.00)	-0.32	(−1.22,−0.01)	-0.19	(−1.10,0.00)	-0.41	(−1.25,0.00)	-0.40	(−1.57,0.00)	-0.48	(−0.98,−0.17)	0.00	(0.00,0.00)
$\sum_{\beta>0.01}$	9.90	(6.87,13.90)	1.91	(1.23,2.92)	2.35	(1.64,3.61)	2.17	(1.40,3.25)	2.77	(1.86,4.09)	2.75	(1.75,4.25)	2.63	(1.98,3.58)	2.85	(2.05,3.63)
$P_{\beta<-0.01}$	0.44	(0.35,0.55)	0.03	(0.00,0.10)	0.15	(0.03,0.30)	0.08	(0.00,0.25)	0.16	(0.00,0.35)	0.16	(0.00,0.40)	0.21	(0.10,0.33)	0.00	(0.00,0.00)
$P_{\beta>0.01}$	0.54	(0.42,0.62)	0.46	(0.33,0.62)	0.76	(0.62,0.90)	0.64	(0.33,0.88)	0.76	(0.55,0.97)	0.77	(0.55,0.97)	0.72	(0.57,0.85)	0.34	(0.20,0.45)
Intercept	-3.08	(−3.81,−2.62)	-2.42	(−2.82,−2.09)	-2.43	(−2.92,−2.09)	-2.43	(−2.84,−2.10)	-2.67	(−3.20,−2.31)	-2.65	(−3.20,−2.26)	-2.58	(−3.02,−2.25)	-2.79	(−3.33,−2.42)
AGE	0.02	(−0.48,0.43)	-0.00	(−0.13,0.12)	0.00	(−0.11,0.13)	-0.00	(−0.13,0.13)	0.01	(−0.26,0.32)	0.01	(−0.21,0.34)	0.01	(−0.16,0.18)	0.06	(−0.27,0.35)
Subm.L.Dm	0.14	(−1.82,1.98)	0.06	(−0.00,0.38)	0.07	(−0.03,0.17)	0.07	(−0.00,0.26)	0.07	(−0.05,0.19)	0.06	(−0.07,0.16)	0.08	(−0.04,0.21)	0.04	(0.00,0.31)
Subm.L.V10	0.03	(−1.37,1.09)	0.02	(−0.01,0.23)	0.01	(−0.11,0.17)	0.02	(−0.08,0.18)	-0.00	(−0.11,0.08)	-0.00	(−0.11,0.08)	0.02	(−0.17,0.16)	0.05	(0.00,0.43)
Subm.L.V30	0.02	(−1.01,1.41)	0.02	(−0.01,0.15)	0.02	(−0.08,0.15)	0.03	(−0.08,0.15)	0.01	(−0.15,0.14)	0.01	(−0.16,0.13)	0.03	(−0.12,0.17)	0.02	(0.00,0.24)
Subm.L.V50	0.03	(−0.68,0.75)	0.06	(−0.01,0.35)	0.07	(−0.06,0.24)	0.07	(−0.03,0.35)	0.09	(−0.06,0.24)	0.08	(−0.09,0.25)	0.07	(−0.07,0.23)	0.09	(0.00,0.47)
Subm.R.Dm	0.09	(−1.66,1.43)	0.07	(−0.01,0.31)	0.06	(−0.06,0.20)	0.06	(−0.04,0.22)	0.08	(−0.06,0.19)	0.07	(−0.11,0.19)	0.07	(−0.06,0.21)	0.06	(0.00,0.33)
Subm.R.V10	-0.00	(−1.15,0.98)	0.01	(−0.01,0.14)	0.02	(−0.12,0.10)	0.02	(−0.11,0.11)	0.01	(−0.07,0.08)	0.01	(−0.07,0.09)	0.02	(−0.12,0.13)	0.03	(0.00,0.33)
Subm.R.V30	-0.04	(−1.18,0.96)	0.02	(−0.01,0.15)	0.02	(−0.09,0.11)	0.02	(−0.08,0.11)	0.02	(−0.08,0.09)	0.02	(−0.10,0.09)	0.02	(−0.14,0.14)	0.03	(0.00,0.23)
Subm.R.V50	0.06	(−0.69,0.64)	0.04	(−0.01,0.33)	0.06	(−0.10,0.17)	0.05	(−0.10,0.20)	0.08	(−0.11,0.23)	0.08	(−0.11,0.22)	0.07	(−0.09,0.19)	0.05	(0.00,0.35)
Parotid.L.Dm	-0.05	(−1.52,1.57)	0.02	(−0.01,0.24)	0.03	(−0.06,0.11)	0.03	(−0.07,0.15)	0.03	(−0.12,0.14)	0.04	(−0.13,0.14)	0.04	(−0.13,0.15)	0.03	(0.00,0.23)
Parotid.L.V10	0.06	(−0.66,0.70)	0.03	(−0.00,0.22)	0.05	(−0.06,0.16)	0.05	(−0.01,0.19)	0.06	(−0.09,0.17)	0.06	(−0.05,0.17)	0.06	(−0.11,0.19)	0.05	(0.00,0.35)
Parotid.L.V30	0.09	(−1.08,1.33)	0.05	(−0.01,0.31)	0.05	(−0.06,0.17)	0.05	(−0.03,0.19)	0.05	(−0.08,0.14)	0.05	(−0.08,0.14)	0.04	(−0.10,0.20)	0.04	(0.00,0.36)
Parotid.L.V50	0.07	(−1.08,1.12)	0.05	(−0.01,0.29)	0.06	(−0.06,0.20)	0.05	(−0.04,0.24)	0.06	(−0.11,0.23)	0.07	(−0.16,0.28)	0.07	(−0.09,0.21)	0.07	(0.00,0.37)
Parotid.R.Dm	0.12	(−1.67,1.89)	0.04	(−0.01,0.26)	0.05	(−0.05,0.16)	0.05	(−0.01,0.16)	0.05	(−0.10,0.20)	0.06	(−0.14,0.19)	0.06	(−0.07,0.18)	0.04	(0.00,0.32)
Parotid.R.V10	0.03	(−0.95,0.76)	0.04	(−0.01,0.24)	0.04	(−0.10,0.16)	0.04	(−0.06,0.16)	0.04	(−0.09,0.13)	0.05	(−0.05,0.13)	0.05	(−0.09,0.19)	0.05	(0.00,0.34)
Parotid.R.V30	0.03	(−1.62,1.34)	0.05	(−0.01,0.30)	0.05	(−0.04,0.16)	0.06	(−0.01,0.21)	0.06	(−0.03,0.15)	0.07	(−0.04,0.17)	0.06	(−0.07,0.19)	0.03	(0.00,0.24)
Parotid.R.V50	0.02	(−1.15,1.47)	0.05	(−0.01,0.39)	0.07	(−0.03,0.20)	0.07	(−0.01,0.25)	0.07	(−0.10,0.23)	0.08	(−0.11,0.22)	0.06	(−0.07,0.18)	0.08	(0.00,0.49)
PCM.Sup.Dm	0.04	(−1.18,1.54)	0.04	(−0.01,0.26)	0.07	(−0.06,0.18)	0.06	(−0.01,0.19)	0.09	(−0.01,0.24)	0.09	(−0.01,0.21)	0.06	(−0.06,0.19)	0.11	(0.00,0.56)
PCM.Sup.V10	-0.01	(−1.12,1.21)	0.03	(−0.01,0.32)	0.03	(−0.06,0.12)	0.03	(−0.04,0.21)	0.04	(−0.06,0.13)	0.03	(−0.09,0.09)	0.03	(−0.10,0.14)	0.07	(0.00,0.60)
PCM.Sup.V30	0.11	(−0.72,1.08)	0.05	(−0.00,0.32)	0.06	(−0.04,0.16)	0.06	(−0.01,0.24)	0.07	(−0.00,0.17)	0.07	(0.00,0.16)	0.05	(−0.06,0.17)	0.05	(0.00,0.32)
PCM.Sup.V50	0.16	(−0.86,1.09)	0.08	(−0.00,0.51)	0.09	(−0.01,0.24)	0.09	(−0.01,0.28)	0.12	(0.02,0.27)	0.12	(0.02,0.26)	0.09	(−0.04,0.25)	0.14	(0.00,0.71)
PCM.Med.Dm	0.22	(−0.66,1.07)	0.13	(−0.01,0.46)	0.09	(−0.03,0.24)	0.10	(−0.01,0.38)	0.10	(−0.06,0.23)	0.11	(−0.06,0.30)	0.11	(−0.03,0.25)	0.11	(0.00,0.49)
PCM.Med.V10	0.02	(−0.76,0.85)	0.03	(−0.02,0.24)	0.04	(−0.09,0.20)	0.04	(−0.10,0.23)	0.02	(−0.09,0.17)	0.03	(−0.08,0.21)	0.05	(−0.11,0.21)	0.06	(0.00,0.41)
PCM.Med.V30	0.12	(−0.69,0.97)	0.05	(−0.01,0.25)	0.05	(−0.06,0.14)	0.05	(−0.05,0.17)	0.02	(−0.10,0.10)	0.03	(−0.11,0.10)	0.06	(−0.07,0.19)	0.06	(0.00,0.39)
PCM.Med.V50	0.02	(−0.66,0.74)	0.06	(−0.00,0.35)	0.06	(−0.07,0.22)	0.06	(−0.02,0.25)	0.10	(−0.10,0.26)	0.10	(−0.12,0.27)	0.06	(−0.10,0.20)	0.06	(0.00,0.38)
PCM.Inf.Dm	0.30	(−0.35,0.95)	0.08	(−0.01,0.35)	0.10	(−0.03,0.25)	0.09	(−0.02,0.26)	0.10	(−0.01,0.30)	0.11	(−0.03,0.31)	0.10	(−0.09,0.26)	0.14	(0.00,0.48)
PCM.Inf.V10	0.10	(−0.66,1.00)	0.01	(−0.14,0.17)	0.02	(−0.14,0.16)	0.02	(−0.14,0.13)	0.02	(−0.13,0.16)	0.01	(−0.12,0.15)	0.02	(−0.15,0.20)	0.04	(0.00,0.26)
PCM.Inf.V30	-0.10	(−0.56,0.48)	0.00	(−0.11,0.14)	-0.01	(−0.17,0.11)	-0.00	(−0.16,0.12)	0.00	(−0.19,0.18)	0.01	(−0.27,0.18)	-0.02	(−0.16,0.16)	0.02	(0.00,0.17)
PCM.Inf.V50	0.09	(−0.62,0.96)	0.05	(−0.01,0.47)	0.06	(−0.06,0.23)	0.06	(−0.02,0.38)	0.10	(−0.01,0.29)	0.10	(−0.03,0.32)	0.06	(−0.07,0.25)	0.12	(0.00,0.68)
Supraglottic.Dm	0.12	(−0.57,0.83)	0.07	(−0.01,0.31)	0.07	(−0.08,0.21)	0.07	(−0.03,0.27)	0.10	(−0.06,0.32)	0.09	(−0.06,0.31)	0.08	(−0.06,0.24)	0.10	(0.00,0.41)
Supraglottic.V10	0.02	(−0.74,0.77)	0.03	(−0.01,0.24)	0.03	(−0.12,0.16)	0.03	(−0.03,0.18)	0.04	(−0.11,0.24)	0.04	(−0.11,0.26)	0.02	(−0.16,0.15)	0.04	(0.00,0.34)
Supraglottic.V30	-0.02	(−0.75,0.65)	0.02	(−0.02,0.26)	0.03	(−0.15,0.16)	0.03	(−0.14,0.26)	0.03	(−0.12,0.17)	0.03	(−0.13,0.18)	0.03	(−0.16,0.27)	0.02	(0.00,0.22)
Supraglottic.V50	0.12	(−0.52,0.68)	0.07	(−0.01,0.33)	0.08	(−0.02,0.24)	0.07	(−0.01,0.28)	0.12	(0.00,0.26)	0.11	(0.00,0.26)	0.10	(−0.03,0.26)	0.09	(0.00,0.40)
OralCavity.Ext.Dm	0.23	(−1.26,2.17)	0.17	(−0.00,0.65)	0.13	(0.03,0.30)	0.13	(0.00,0.44)	0.15	(0.04,0.35)	0.14	(0.04,0.34)	0.13	(−0.00,0.28)	0.15	(0.00,0.71)
OralCavity.Ext.V10	0.04	(−1.29,1.28)	0.01	(−0.01,0.19)	0.04	(−0.05,0.14)	0.03	(−0.03,0.14)	0.04	(−0.03,0.12)	0.05	(−0.04,0.11)	0.04	(−0.06,0.15)	0.03	(0.00,0.28)
OralCavity.Ext.V30	0.15	(−1.11,1.27)	0.08	(−0.00,0.45)	0.08	(−0.02,0.21)	0.08	(−0.02,0.26)	0.10	(0.02,0.23)	0.10	(0.03,0.24)	0.08	(−0.06,0.22)	0.14	(0.00,0.62)
OralCavity.Ext.V50	0.34	(−1.54,2.33)	0.14	(−0.00,0.57)	0.13	(−0.01,0.29)	0.14	(−0.00,0.49)	0.15	(0.01,0.31)	0.15	(0.01,0.39)	0.14	(−0.01,0.31)	0.31	(0.00,0.82)
GlotticArea.Dm	0.12	(−0.66,0.84)	0.02	(−0.01,0.18)	0.03	(−0.07,0.18)	0.03	(−0.05,0.19)	0.02	(−0.09,0.18)	0.02	(−0.12,0.15)	0.03	(−0.15,0.18)	0.11	(0.00,0.50)
GlotticArea.V10	0.01	(−0.81,0.64)	0.01	(−0.04,0.15)	0.02	(−0.11,0.17)	0.02	(−0.09,0.21)	0.02	(−0.11,0.18)	0.02	(−0.10,0.18)	0.02	(−0.12,0.15)	0.03	(0.00,0.26)
GlotticArea.V30	-0.12	(−0.64,0.41)	-0.00	(−0.17,0.13)	-0.02	(−0.17,0.14)	-0.02	(−0.18,0.15)	-0.04	(−0.33,0.12)	-0.04	(−0.33,0.15)	-0.02	(−0.17,0.15)	0.02	(0.00,0.22)
GlotticArea.V50	0.03	(−0.81,0.81)	0.02	(−0.02,0.17)	0.03	(−0.09,0.15)	0.02	(−0.06,0.18)	0.05	(−0.08,0.18)	0.04	(−0.07,0.20)	0.03	(−0.11,0.18)	0.11	(0.00,0.52)
DYSFAGIE..BSL.2	0.33	(−0.09,0.63)	0.15	(−0.01,0.45)	0.14	(−0.01,0.34)	0.14	(−0.00,0.42)	0.24	(−0.04,0.63)	0.20	(−0.09,0.65)	0.14	(−0.04,0.34)	0.29	(−0.08,0.66)
DYSFAGIE..BSL.3	0.59	(0.25,0.97)	0.34	(0.03,0.64)	0.26	(0.09,0.49)	0.30	(0.05,0.61)	0.43	(0.00,0.87)	0.38	(−0.00,0.89)	0.28	(0.09,0.51)	0.54	(0.24,0.90)

Table 8 Mean model coefficients for $D_{\triangle}$.

	LR		Lasso		Ridge		ElasticNet		PCLR		LAELR		Dropout		LRnn	
$\sum_{\beta<-0.01}$	-12.37	(−17.94,−7.79)	-0.05	(−0.62,0.00)	-0.15	(−0.90,0.00)	-0.12	(−0.75,0.00)	-0.26	(−1.67,0.00)	-0.27	(−1.70,0.00)	-0.38	(−1.11,−0.09)	0.00	(0.00,0.00)
$\sum_{\beta>0.01}$	14.95	(10.05,19.91)	1.75	(1.16,2.67)	2.04	(1.38,3.15)	1.97	(1.33,3.10)	2.46	(1.75,4.65)	2.50	(1.70,4.57)	2.43	(1.86,3.48)	2.57	(1.85,3.63)
$P_{\beta<-0.01}$	0.46	(0.38,0.55)	0.02	(0.00,0.10)	0.08	(0.00,0.25)	0.06	(0.00,0.23)	0.10	(0.00,0.30)	0.10	(0.00,0.38)	0.18	(0.05,0.33)	0.00	(0.00,0.00)
$P_{\beta>0.01}$	0.53	(0.45,0.60)	0.46	(0.28,0.68)	0.84	(0.68,0.97)	0.71	(0.28,0.95)	0.82	(0.57,1.00)	0.83	(0.57,1.00)	0.74	(0.60,0.88)	0.27	(0.15,0.38)
Intercept	-3.02	(−3.63,−2.57)	-2.44	(−2.89,−2.09)	-2.46	(−2.86,−2.12)	-2.47	(−2.84,−2.14)	-2.66	(−3.16,−2.29)	-2.65	(−3.13,−2.26)	-2.61	(−3.01,−2.27)	-2.77	(−3.33,−2.40)
AGE	0.01	(−0.32,0.37)	-0.01	(−0.16,0.11)	-0.02	(−0.15,0.13)	-0.02	(−0.15,0.12)	-0.02	(−0.29,0.27)	-0.01	(−0.21,0.24)	-0.02	(−0.22,0.15)	0.01	(−0.31,0.35)
Subm.L.Dm	0.00	(−2.43,1.84)	0.04	(−0.00,0.36)	0.05	(0.01,0.12)	0.05	(−0.00,0.14)	0.05	(−0.02,0.12)	0.05	(−0.01,0.11)	0.06	(−0.04,0.17)	0.03	(0.00,0.32)
Subm.L.V10	0.10	(−1.79,2.57)	0.02	(−0.01,0.16)	0.03	(−0.02,0.08)	0.03	(−0.02,0.09)	0.02	(−0.05,0.07)	0.02	(−0.06,0.08)	0.03	(−0.10,0.15)	0.04	(0.00,0.45)
Subm.L.V30	-0.05	(−2.33,1.97)	0.02	(−0.00,0.11)	0.03	(−0.04,0.09)	0.02	(−0.03,0.09)	0.02	(−0.16,0.08)	0.02	(−0.16,0.10)	0.02	(−0.09,0.14)	0.03	(0.00,0.41)
Subm.L.V50	0.08	(−1.33,1.25)	0.06	(−0.00,0.41)	0.06	(−0.03,0.14)	0.07	(−0.02,0.25)	0.07	(−0.01,0.19)	0.06	(−0.14,0.18)	0.07	(−0.09,0.19)	0.07	(0.00,0.49)
Subm.R.Dm	0.10	(−2.14,1.77)	0.06	(−0.00,0.60)	0.06	(−0.02,0.14)	0.05	(−0.00,0.17)	0.07	(0.02,0.29)	0.07	(0.02,0.25)	0.06	(−0.03,0.18)	0.05	(0.00,0.62)
Subm.R.V10	0.01	(−2.02,1.96)	0.01	(−0.00,0.03)	0.03	(−0.05,0.10)	0.02	(−0.04,0.10)	0.02	(−0.04,0.17)	0.02	(−0.04,0.09)	0.03	(−0.11,0.14)	0.01	(0.00,0.15)
Subm.R.V30	-0.10	(−2.10,1.65)	0.02	(−0.01,0.17)	0.03	(−0.07,0.12)	0.03	(−0.05,0.16)	0.03	(−0.04,0.22)	0.03	(−0.05,0.12)	0.02	(−0.12,0.16)	0.03	(0.00,0.43)
Subm.R.V50	0.14	(−1.14,1.39)	0.10	(−0.00,0.49)	0.07	(−0.01,0.20)	0.09	(0.00,0.38)	0.09	(0.03,0.23)	0.09	(−0.10,0.31)	0.08	(−0.05,0.25)	0.11	(0.00,0.60)
Parotid.L.Dm	0.18	(−1.88,2.23)	0.03	(−0.00,0.30)	0.05	(−0.00,0.11)	0.05	(−0.00,0.11)	0.06	(−0.04,0.14)	0.07	(−0.06,0.15)	0.05	(−0.07,0.16)	0.01	(0.00,0.23)
Parotid.L.V10	0.14	(−1.09,1.49)	0.06	(−0.00,0.41)	0.05	(−0.08,0.12)	0.05	(−0.05,0.15)	0.04	(−0.30,0.10)	0.05	(−0.18,0.11)	0.06	(−0.07,0.18)	0.06	(0.00,0.54)
Parotid.L.V30	-0.11	(−1.98,2.00)	0.05	(−0.00,0.27)	0.05	(−0.02,0.12)	0.04	(−0.01,0.17)	0.06	(−0.06,0.15)	0.07	(−0.08,0.16)	0.05	(−0.06,0.17)	0.04	(0.00,0.29)
Parotid.L.V50	0.07	(−1.25,1.28)	0.04	(−0.01,0.35)	0.05	(−0.05,0.16)	0.05	(−0.04,0.24)	0.09	(−0.08,0.36)	0.09	(−0.12,0.24)	0.06	(−0.08,0.21)	0.09	(0.00,0.42)
Parotid.R.Dm	0.31	(−2.32,1.94)	0.03	(−0.00,0.20)	0.06	(0.01,0.13)	0.05	(−0.00,0.13)	0.08	(−0.05,0.17)	0.09	(−0.09,0.18)	0.06	(−0.04,0.15)	0.01	(0.00,0.18)
Parotid.R.V10	-0.08	(−1.72,1.23)	0.05	(−0.01,0.39)	0.05	(−0.12,0.14)	0.04	(−0.11,0.17)	0.06	(−0.07,0.11)	0.06	(−0.06,0.12)	0.05	(−0.07,0.15)	0.06	(0.00,0.48)
Parotid.R.V30	-0.04	(−1.57,1.82)	0.06	(−0.00,0.38)	0.06	(0.01,0.14)	0.06	(−0.00,0.18)	0.08	(−0.07,0.18)	0.09	(−0.07,0.18)	0.06	(−0.04,0.18)	0.04	(0.00,0.36)
Parotid.R.V50	0.02	(−1.09,1.26)	0.07	(−0.01,0.45)	0.07	(−0.04,0.21)	0.07	(−0.00,0.20)	0.10	(−0.07,0.20)	0.10	(−0.10,0.23)	0.08	(−0.08,0.24)	0.12	(0.00,0.53)
PCM.Sup.Dm	0.20	(−2.79,2.57)	0.02	(−0.00,0.10)	0.06	(0.02,0.11)	0.05	(0.00,0.10)	0.07	(0.03,0.11)	0.07	(0.02,0.16)	0.06	(−0.03,0.17)	0.02	(0.00,0.26)
PCM.Sup.V10	-0.24	(−2.36,1.94)	0.02	(−0.01,0.20)	0.03	(−0.04,0.10)	0.03	(−0.03,0.10)	0.03	(−0.05,0.07)	0.03	(−0.03,0.11)	0.03	(−0.08,0.11)	0.03	(0.00,0.48)
PCM.Sup.V30	0.12	(−1.57,2.01)	0.04	(−0.00,0.34)	0.05	(0.01,0.12)	0.04	(−0.00,0.11)	0.05	(−0.03,0.10)	0.06	(0.01,0.10)	0.05	(−0.04,0.16)	0.07	(0.00,0.70)
PCM.Sup.V50	-0.04	(−1.30,1.34)	0.05	(−0.00,0.40)	0.07	(0.01,0.17)	0.07	(−0.00,0.19)	0.10	(0.01,0.19)	0.10	(−0.00,0.20)	0.08	(−0.04,0.20)	0.11	(0.00,0.67)
PCM.Med.Dm	0.18	(−2.08,1.98)	0.12	(−0.00,0.58)	0.07	(0.02,0.14)	0.08	(−0.00,0.25)	0.08	(0.03,0.17)	0.08	(0.02,0.18)	0.07	(−0.05,0.22)	0.04	(0.00,0.44)
PCM.Med.V10	0.08	(−1.45,1.35)	0.03	(−0.01,0.34)	0.04	(−0.04,0.13)	0.04	(−0.02,0.25)	0.02	(−0.05,0.11)	0.02	(−0.06,0.14)	0.05	(−0.04,0.16)	0.08	(0.00,0.50)
PCM.Med.V30	0.14	(−1.74,1.50)	0.03	(−0.00,0.30)	0.04	(−0.03,0.12)	0.04	(−0.01,0.12)	0.04	(−0.02,0.08)	0.04	(−0.02,0.10)	0.05	(−0.05,0.18)	0.04	(0.00,0.53)
PCM.Med.V50	-0.01	(−1.47,1.02)	0.12	(−0.00,0.53)	0.07	(−0.05,0.19)	0.09	(−0.05,0.33)	0.12	(0.02,0.26)	0.10	(−0.11,0.29)	0.08	(−0.05,0.24)	0.12	(0.00,0.55)
PCM.Inf.Dm	0.07	(−1.81,1.54)	0.02	(−0.01,0.18)	0.05	(−0.02,0.13)	0.04	(−0.02,0.16)	0.06	(−0.00,0.16)	0.05	(−0.02,0.24)	0.06	(−0.07,0.24)	0.07	(0.00,0.53)
PCM.Inf.V10	0.04	(−1.12,1.37)	0.02	(−0.02,0.15)	0.02	(−0.07,0.12)	0.02	(−0.05,0.12)	0.00	(−0.10,0.15)	0.00	(−0.11,0.13)	0.01	(−0.09,0.15)	0.02	(0.00,0.30)
PCM.Inf.V30	-0.04	(−0.92,0.85)	0.01	(−0.02,0.20)	0.01	(−0.10,0.10)	0.01	(−0.11,0.13)	0.04	(−0.21,0.13)	0.04	(−0.21,0.13)	0.02	(−0.17,0.16)	0.02	(0.00,0.21)
PCM.Inf.V50	0.15	(−1.35,1.76)	0.04	(−0.02,0.38)	0.05	(−0.05,0.17)	0.05	(−0.02,0.14)	0.05	(−0.02,0.23)	0.06	(−0.02,0.23)	0.05	(−0.08,0.24)	0.10	(0.00,0.55)
Supraglottic.Dm	0.05	(−1.93,2.55)	0.06	(−0.01,0.35)	0.07	(0.00,0.14)	0.06	(−0.00,0.28)	0.08	(−0.02,0.20)	0.08	(0.02,0.23)	0.07	(−0.05,0.21)	0.07	(0.00,0.43)
Supraglottic.V10	0.04	(−1.32,1.39)	0.04	(−0.01,0.24)	0.03	(−0.06,0.15)	0.03	(−0.04,0.23)	0.01	(−0.13,0.17)	0.01	(−0.06,0.21)	0.03	(−0.14,0.16)	0.05	(0.00,0.41)
Supraglottic.V30	-0.07	(−1.22,1.15)	0.03	(−0.01,0.27)	0.02	(−0.07,0.12)	0.02	(−0.09,0.15)	0.04	(−0.22,0.15)	0.04	(−0.23,0.17)	0.03	(−0.13,0.14)	0.03	(0.00,0.36)
Supraglottic.V50	0.24	(−1.28,1.72)	0.07	(−0.00,0.35)	0.08	(0.01,0.20)	0.08	(−0.00,0.32)	0.09	(0.01,0.21)	0.09	(0.02,0.23)	0.09	(−0.02,0.25)	0.10	(0.00,0.43)
OralCavity.Ext.Dm	0.34	(−1.43,2.39)	0.08	(−0.00,0.54)	0.08	(0.04,0.15)	0.09	(0.00,0.37)	0.09	(0.04,0.26)	0.08	(0.01,0.28)	0.09	(−0.01,0.19)	0.05	(0.00,0.60)
OralCavity.Ext.V10	0.07	(−2.63,2.08)	0.03	(−0.01,0.29)	0.04	(−0.07,0.10)	0.04	(−0.07,0.19)	0.03	(−0.02,0.10)	0.04	(−0.02,0.14)	0.04	(−0.11,0.14)	0.11	(0.00,0.78)
OralCavity.Ext.V30	0.10	(−1.64,1.66)	0.07	(−0.00,0.58)	0.07	(0.02,0.15)	0.07	(0.00,0.20)	0.08	(0.04,0.19)	0.08	(0.04,0.21)	0.07	(−0.05,0.19)	0.12	(0.00,0.87)
OralCavity.Ext.V50	0.26	(−0.97,1.22)	0.15	(−0.00,0.60)	0.11	(0.03,0.27)	0.12	(0.00,0.35)	0.13	(0.05,0.41)	0.13	(0.04,0.42)	0.13	(−0.01,0.29)	0.33	(0.00,0.83)
GlotticArea.Dm	0.19	(−2.04,2.06)	0.00	(−0.01,0.04)	0.01	(−0.05,0.09)	0.01	(−0.03,0.09)	0.02	(−0.05,0.12)	0.02	(−0.07,0.13)	0.00	(−0.11,0.11)	0.05	(0.00,0.43)
GlotticArea.V10	0.05	(−1.12,1.11)	0.01	(−0.03,0.12)	0.02	(−0.08,0.11)	0.02	(−0.07,0.13)	0.01	(−0.11,0.20)	0.01	(−0.11,0.19)	0.03	(−0.11,0.21)	0.03	(0.00,0.23)
GlotticArea.V30	-0.09	(−0.79,0.78)	-0.01	(−0.15,0.03)	-0.01	(−0.12,0.08)	-0.02	(−0.15,0.07)	0.00	(−0.19,0.12)	0.00	(−0.37,0.11)	-0.02	(−0.17,0.14)	0.01	(0.00,0.14)
GlotticArea.V50	-0.04	(−1.46,1.05)	0.01	(−0.02,0.12)	0.02	(−0.07,0.13)	0.02	(−0.04,0.13)	0.03	(−0.06,0.23)	0.03	(−0.07,0.27)	0.02	(−0.14,0.18)	0.10	(0.00,0.46)
DYSFAGIE..BSL.2	0.20	(−0.14,0.54)	0.08	(−0.01,0.33)	0.08	(−0.02,0.25)	0.09	(−0.01,0.29)	0.10	(−0.13,0.52)	0.08	(−0.11,0.47)	0.11	(−0.07,0.28)	0.18	(−0.13,0.51)
DYSFAGIE..BSL.3	0.36	(0.03,0.74)	0.19	(−0.00,0.48)	0.15	(0.04,0.36)	0.17	(0.04,0.44)	0.21	(0.02,0.61)	0.15	(0.02,0.55)	0.19	(0.01,0.41)	0.34	(0.04,0.65)

Table 9 Mean model coefficients for A*.

	LR		Lasso		Ridge		ElasticNet		PCLR		LAELR		Dropout		LRnn	
$\sum_{\beta_{<-0.01}}$	-0.08	(−0.09,−0.06)	0.00	(0.00,0.00)	0.00	(0.00,0.00)	0.00	(0.00,0.00)	0.00	(0.00,0.00)	0.00	(0.00,0.00)	0.00	(0.00,0.00)	0.00	(0.00,0.00)
$\sum_{\beta_{>0.01}}$	1.78	(1.76,1.79)	1.36	(1.31,1.41)	1.34	(1.31,1.38)	1.35	(1.29,1.40)	1.72	(1.69,1.74)	1.72	(1.69,1.74)	1.26	(1.17,1.37)	1.72	(1.70,1.73)
$P_{\beta_{<-0.01}}$	0.25	(0.25,0.25)	0.00	(0.00,0.00)	0.00	(0.00,0.00)	0.00	(0.00,0.00)	0.00	(0.00,0.00)	0.00	(0.00,0.00)	0.00	(0.00,0.00)	0.00	(0.00,0.00)
$P_{\beta_{>0.01}}$	0.75	(0.75,0.75)	0.92	(0.75,1.00)	1.00	(1.00,1.00)	1.00	(1.00,1.00)	1.00	(1.00,1.00)	1.00	(1.00,1.00)	1.00	(1.00,1.00)	0.94	(0.75,1.00)
Intercept	-1.51	(−1.53,−1.49)	-1.35	(−1.37,−1.33)	-1.34	(−1.36,−1.33)	-1.34	(−1.37,−1.31)	-1.52	(−1.54,−1.51)	-1.52	(−1.54,−1.44)	-1.34	(−1.38,−1.31)	-1.52	(−1.53,−1.50)
AGE	-0.02	(−0.02,−0.02)	-0.01	(−0.03,−0.00)	-0.04	(−0.05,−0.04)	-0.02	(−0.04,−0.01)	-0.02	(−0.03,−0.02)	-0.02	(−0.03,−0.02)	-0.05	(−0.08,−0.02)	-0.02	(−0.02,−0.01)
Subm.L.Dm	-0.08	(−0.09,−0.06)	0.02	(0.01,0.05)	0.20	(0.16,0.21)	0.12	(0.06,0.17)	0.37	(0.25,0.46)	0.34	(0.17,0.46)	0.25	(0.22,0.29)	0.03	(0.00,0.08)
Subm.R.Dm	0.85	(0.84,0.86)	0.51	(0.48,0.55)	0.37	(0.36,0.46)	0.42	(0.38,0.47)	0.50	(0.43,0.63)	0.53	(0.44,0.69)	0.38	(0.34,0.44)	0.78	(0.75,0.81)
Parotid.L.Dm	0.58	(0.57,0.59)	0.46	(0.45,0.47)	0.37	(0.36,0.40)	0.41	(0.38,0.43)	0.44	(0.37,0.49)	0.44	(0.38,0.52)	0.30	(0.27,0.34)	0.53	(0.51,0.55)
Parotid.R.Dm	0.34	(0.33,0.35)	0.38	(0.37,0.39)	0.40	(0.39,0.41)	0.40	(0.38,0.42)	0.41	(0.35,0.46)	0.40	(0.33,0.45)	0.33	(0.28,0.36)	0.37	(0.36,0.38)
XER.BSL.2	0.34	(0.33,0.34)	0.24	(0.23,0.26)	0.26	(0.25,0.27)	0.25	(0.23,0.27)	0.32	(0.31,0.33)	0.32	(0.31,0.33)	0.18	(0.15,0.22)	0.33	(0.33,0.34)
XER.BSL.3	0.34	(0.07,0.85)	0.06	(0.04,0.07)	0.09	(0.09,0.11)	0.08	(0.06,0.09)	0.22	(0.08,0.65)	0.39	(−0.01,2.38)	0.14	(−0.01,0.26)	0.32	(0.09,0.75)

Table 10 Mean model coefficients for $B^*_{\triangle}$.

	LR		Lasso		Ridge		ElasticNet		PCLR		LAELR		Dropout		LRnn	
$\sum_{\beta_{<-0.01}}$	-3.08	(−3.44,−2.77)	-0.00	(0.00,0.00)	0.00	(0.00,0.00)	0.00	(0.00,0.00)	0.00	(0.00,0.00)	-0.02	(−0.22,0.00)	-0.00	(−0.03,0.00)	0.00	(0.00,0.00)
$\sum_{\beta_{>0.01}}$	5.06	(4.74,5.53)	1.37	(1.29,1.47)	1.50	(1.47,1.54)	1.49	(1.41,1.55)	2.10	(2.05,2.16)	2.13	(2.03,2.43)	1.69	(1.63,1.76)	2.23	(2.14,2.65)
$P_{\beta_{<-0.01}}$	0.47	(0.44,0.50)	0.00	(0.00,0.00)	0.00	(0.00,0.00)	0.00	(0.00,0.00)	0.00	(0.00,0.00)	0.02	(0.00,0.25)	0.00	(0.00,0.06)	0.00	(0.00,0.00)
$P_{\beta_{>0.01}}$	0.53	(0.50,0.56)	0.79	(0.69,0.88)	1.00	(1.00,1.00)	0.99	(0.88,1.00)	1.00	(1.00,1.00)	0.97	(0.69,1.00)	0.98	(0.94,1.00)	0.77	(0.69,0.88)
Intercept	-1.80	(−1.97,−1.74)	-1.36	(−1.42,−1.33)	-1.39	(−1.41,−1.37)	-1.39	(−1.42,−1.35)	-1.67	(−1.72,−1.65)	-1.69	(−1.75,−1.65)	-1.50	(−1.52,−1.47)	-1.77	(−1.99,−1.72)
AGE	-0.05	(−0.06,−0.05)	-0.01	(−0.03,−0.00)	-0.03	(−0.03,−0.02)	-0.02	(−0.04,−0.01)	0.04	(−0.01,0.07)	0.04	(0.00,0.06)	-0.03	(−0.06,0.01)	-0.04	(−0.04,−0.03)
Subm.L.Dm	-0.29	(−0.33,−0.26)	0.01	(0.00,0.03)	0.06	(0.05,0.06)	0.04	(0.03,0.06)	0.14	(0.11,0.16)	0.14	(−0.03,0.17)	0.07	(0.03,0.11)	0.00	(0.00,0.03)
Subm.L.V10	0.62	(0.51,0.81)	0.02	(0.00,0.08)	0.08	(0.07,0.08)	0.07	(0.04,0.08)	0.16	(0.14,0.19)	0.19	(0.14,0.33)	0.12	(0.08,0.15)	0.10	(0.02,0.28)
Subm.L.V30	-0.71	(−0.76,−0.65)	0.00	(−0.00,0.01)	0.02	(0.01,0.03)	0.02	(0.00,0.02)	0.15	(0.11,0.18)	0.14	(−0.04,0.18)	0.02	(−0.03,0.06)	0.00	(0.00,0.00)
Subm.L.V50	0.39	(0.37,0.41)	0.05	(0.02,0.08)	0.07	(0.07,0.08)	0.07	(0.05,0.08)	0.12	(0.06,0.14)	0.13	(0.08,0.20)	0.08	(0.04,0.11)	0.06	(0.03,0.10)
Subm.R.Dm	0.26	(0.23,0.29)	0.13	(0.07,0.20)	0.12	(0.11,0.12)	0.12	(0.10,0.15)	0.17	(0.16,0.20)	0.18	(0.14,0.27)	0.15	(0.11,0.19)	0.18	(0.12,0.26)
Subm.R.V10	0.01	(−0.14,0.17)	0.01	(0.00,0.03)	0.09	(0.08,0.10)	0.08	(0.05,0.09)	0.18	(0.16,0.21)	0.17	(0.04,0.20)	0.15	(0.11,0.18)	0.17	(0.08,0.26)
Subm.R.V30	0.57	(0.49,0.72)	0.05	(0.02,0.10)	0.10	(0.09,0.11)	0.10	(0.07,0.12)	0.18	(0.16,0.21)	0.19	(0.16,0.38)	0.16	(0.12,0.20)	0.51	(0.40,0.79)
Subm.R.V50	0.07	(0.05,0.09)	0.16	(0.13,0.19)	0.13	(0.12,0.14)	0.14	(0.12,0.17)	0.16	(0.15,0.19)	0.15	(0.12,0.20)	0.13	(0.10,0.16)	0.14	(0.09,0.18)
Parotid.L.Dm	1.25	(1.09,1.40)	0.13	(0.08,0.18)	0.11	(0.10,0.11)	0.11	(0.08,0.15)	0.10	(0.09,0.11)	0.12	(0.08,0.35)	0.11	(0.07,0.14)	0.15	(0.07,0.22)
Parotid.L.V10	-0.32	(−0.38,−0.28)	0.07	(0.04,0.13)	0.12	(0.11,0.12)	0.11	(0.09,0.14)	0.14	(0.13,0.17)	0.13	(0.03,0.16)	0.13	(0.10,0.16)	0.06	(0.01,0.13)
Parotid.L.V30	0.08	(0.03,0.14)	0.14	(0.10,0.19)	0.11	(0.11,0.12)	0.12	(0.10,0.15)	0.09	(0.08,0.10)	0.09	(0.08,0.11)	0.10	(0.06,0.13)	0.18	(0.12,0.23)
Parotid.L.V50	-0.51	(−0.56,−0.46)	0.01	(0.00,0.02)	0.05	(0.04,0.05)	0.04	(0.02,0.05)	0.04	(0.02,0.06)	0.03	(−0.08,0.05)	0.04	(0.01,0.07)	0.01	(0.00,0.04)
Parotid.R.Dm	1.79	(1.58,2.03)	0.07	(0.03,0.13)	0.13	(0.12,0.13)	0.13	(0.10,0.15)	0.13	(0.12,0.14)	0.15	(0.10,0.46)	0.12	(0.08,0.15)	0.07	(0.01,0.12)
Parotid.R.V10	-0.15	(−0.24,−0.08)	0.39	(0.36,0.42)	0.16	(0.15,0.17)	0.20	(0.16,0.28)	0.15	(0.13,0.20)	0.15	(0.08,0.20)	0.18	(0.14,0.21)	0.47	(0.44,0.50)
Parotid.R.V30	-0.37	(−0.45,−0.29)	0.12	(0.07,0.15)	0.13	(0.13,0.14)	0.13	(0.11,0.15)	0.11	(0.11,0.13)	0.10	(−0.02,0.14)	0.12	(0.09,0.14)	0.13	(0.08,0.17)
Parotid.R.V50	-0.70	(−0.78,−0.62)	0.00	(−0.00,0.01)	0.04	(0.02,0.04)	0.02	(0.01,0.04)	0.07	(0.02,0.10)	0.05	(−0.10,0.10)	0.04	(0.01,0.06)	0.00	(0.00,0.00)
XER.BSL.2	0.36	(0.36,0.36)	0.23	(0.21,0.25)	0.20	(0.18,0.21)	0.21	(0.19,0.24)	0.18	(0.13,0.24)	0.16	(0.12,0.22)	0.16	(0.14,0.20)	0.34	(0.34,0.34)
XER.BSL.3	0.38	(0.13,0.78)	0.05	(0.04,0.07)	0.07	(0.06,0.08)	0.07	(0.05,0.09)	0.04	(−0.20,0.44)	-0.01	(−0.22,0.27)	0.11	(0.03,0.20)	0.42	(0.13,0.91)

Table 11 Mean model coefficients for C*.

	LR		Lasso		Ridge		ElasticNet		PCLR		LAELR		Dropout		LRnn	
$\sum_{\beta_{<-0.01}}$	-0.53	(−0.58,−0.48)	0.00	(0.00,0.00)	0.00	(0.00,0.00)	0.00	(0.00,0.00)	-0.00	(−0.03,0.00)	-0.01	(−0.06,0.00)	-0.00	(0.00,0.00)	0.00	(0.00,0.00)
$\sum_{\beta_{>0.01}}$	3.38	(3.32,3.44)	2.30	(2.22,2.40)	2.26	(2.20,2.32)	2.28	(2.17,2.40)	2.70	(2.54,2.86)	2.76	(2.57,2.96)	2.40	(2.25,2.59)	2.95	(2.90,3.01)
$P_{\beta_{<-0.01}}$	0.33	(0.30,0.40)	0.00	(0.00,0.00)	0.00	(0.00,0.00)	0.00	(0.00,0.00)	0.01	(0.00,0.10)	0.02	(0.00,0.10)	0.00	(0.00,0.00)	0.00	(0.00,0.00)
$P_{\beta_{>0.01}}$	0.55	(0.50,0.70)	0.79	(0.60,1.00)	1.00	(1.00,1.00)	0.97	(0.90,1.00)	0.97	(0.90,1.00)	0.97	(0.90,1.00)	0.99	(0.90,1.00)	0.94	(0.80,1.00)
Intercept	-3.02	(−3.05,−2.99)	-2.69	(−2.75,−2.66)	-2.72	(−2.75,−2.68)	-2.71	(−2.76,−2.64)	-3.02	(−3.10,−2.95)	-3.05	(−3.14,−2.97)	-2.80	(−2.89,−2.73)	-3.04	(−3.08,−3.02)
AGE	0.02	(0.01,0.03)	0.00	(−0.01,0.02)	0.01	(0.00,0.01)	0.00	(−0.01,0.02)	0.05	(0.04,0.07)	0.06	(0.04,0.09)	0.01	(−0.02,0.04)	0.03	(0.02,0.04)
Subm.L.Dm	0.12	(0.08,0.15)	0.03	(0.00,0.10)	0.17	(0.16,0.19)	0.11	(0.05,0.16)	0.29	(0.16,0.39)	0.31	(0.19,0.41)	0.21	(0.16,0.28)	0.11	(0.05,0.18)
Subm.R.Dm	-0.25	(−0.29,−0.22)	0.01	(−0.00,0.04)	0.17	(0.14,0.18)	0.08	(0.02,0.14)	0.32	(0.19,0.45)	0.32	(0.17,0.46)	0.20	(0.13,0.27)	0.05	(0.00,0.14)
Parotid.L.Dm	-0.05	(−0.06,−0.03)	0.04	(0.01,0.06)	0.12	(0.10,0.13)	0.07	(0.04,0.10)	0.21	(0.10,0.30)	0.21	(0.10,0.29)	0.10	(0.06,0.15)	0.02	(0.00,0.05)
Parotid.R.Dm	0.22	(0.20,0.23)	0.15	(0.13,0.16)	0.22	(0.21,0.23)	0.19	(0.17,0.21)	0.26	(0.18,0.31)	0.27	(0.19,0.32)	0.20	(0.17,0.23)	0.13	(0.11,0.15)
PCM.Sup.Dm	-0.22	(−0.25,−0.19)	0.02	(0.00,0.08)	0.27	(0.25,0.28)	0.15	(0.07,0.24)	0.37	(0.27,0.53)	0.33	(0.19,0.50)	0.27	(0.22,0.33)	0.04	(0.00,0.12)
PCM.Med.Dm	0.86	(0.82,0.89)	0.37	(0.31,0.43)	0.27	(0.26,0.29)	0.33	(0.24,0.39)	0.36	(0.27,0.45)	0.40	(0.31,0.52)	0.32	(0.25,0.39)	0.57	(0.50,0.64)
PCM.Inf.Dm	0.57	(0.55,0.59)	0.40	(0.36,0.43)	0.25	(0.23,0.29)	0.32	(0.26,0.37)	0.22	(0.13,0.36)	0.22	(0.13,0.38)	0.24	(0.17,0.30)	0.49	(0.44,0.53)
Supraglottic.Dm	0.01	(−0.01,0.03)	0.15	(0.11,0.20)	0.21	(0.19,0.22)	0.18	(0.15,0.22)	0.24	(0.12,0.31)	0.23	(0.08,0.32)	0.20	(0.15,0.25)	0.09	(0.04,0.16)
OralCavity.Ext.Dm	1.61	(1.58,1.64)	1.15	(1.09,1.19)	0.56	(0.51,0.62)	0.84	(0.65,0.98)	0.40	(0.29,0.64)	0.45	(0.31,0.70)	0.59	(0.46,0.75)	1.37	(1.31,1.41)
GlotticArea.Dm	-0.00	(−0.03,0.03)	0.01	(0.00,0.03)	0.03	(0.02,0.05)	0.02	(0.00,0.04)	0.03	(−0.03,0.13)	0.02	(−0.06,0.12)	0.05	(−0.00,0.09)	0.07	(0.01,0.13)
DYSFAGIE..BSL.2	0.17	(0.17,0.18)	0.16	(0.15,0.17)	0.20	(0.20,0.21)	0.18	(0.17,0.20)	0.22	(0.16,0.24)	0.19	(0.11,0.24)	0.16	(0.13,0.19)	0.19	(0.18,0.19)
DYSFAGIE..BSL.3	0.34	(0.34,0.35)	0.33	(0.33,0.34)	0.36	(0.36,0.37)	0.35	(0.34,0.36)	0.40	(0.36,0.42)	0.38	(0.33,0.42)	0.30	(0.27,0.33)	0.35	(0.35,0.36)

Table 12 Mean model coefficients for $D^{*}_{\triangle}$.

	LR		Lasso		Ridge		ElasticNet		PCLR		LAELR		Dropout		LRnn	
$\sum_{\beta<-0.01}$	-9.10	(−9.73,−8.70)	-0.04	(−0.12,0.00)	-0.15	(−0.18,−0.12)	-0.12	(−0.17,−0.07)	-0.46	(−1.20,−0.08)	-0.30	(−0.93,−0.04)	-0.26	(−0.51,−0.09)	0.00	(0.00,0.00)
$\sum_{\beta>0.01}$	13.39	(12.86,14.06)	1.98	(1.81,2.21)	2.43	(2.38,2.52)	2.29	(2.15,2.45)	4.38	(3.07,5.75)	3.76	(2.61,5.08)	3.23	(2.95,3.62)	4.82	(4.65,4.98)
$P_{\beta<-0.01}$	0.46	(0.42,0.50)	0.03	(0.00,0.07)	0.08	(0.07,0.10)	0.08	(0.05,0.10)	0.20	(0.07,0.30)	0.14	(0.03,0.30)	0.14	(0.10,0.20)	0.00	(0.00,0.00)
$P_{\beta>0.01}$	0.53	(0.50,0.57)	0.42	(0.35,0.53)	0.87	(0.85,0.88)	0.81	(0.65,0.88)	0.71	(0.62,0.85)	0.80	(0.65,0.95)	0.80	(0.70,0.88)	0.50	(0.42,0.57)
Intercept	-3.57	(−3.61,−3.54)	-2.49	(−2.57,−2.42)	-2.61	(−2.63,−2.59)	-2.58	(−2.62,−2.53)	-3.13	(−3.36,−2.93)	-3.04	(−3.32,−2.82)	-2.84	(−2.91,−2.80)	-3.42	(−3.48,−3.38)
AGE	0.08	(0.07,0.09)	0.00	(−0.01,0.01)	0.01	(0.00,0.01)	0.01	(−0.00,0.02)	0.03	(−0.03,0.06)	0.01	(−0.08,0.05)	0.01	(−0.03,0.04)	0.05	(0.04,0.06)
Subm.L.Dm	0.54	(0.49,0.61)	0.03	(0.00,0.11)	0.08	(0.08,0.09)	0.07	(0.05,0.09)	0.05	(0.00,0.10)	0.07	(0.03,0.14)	0.12	(0.07,0.16)	0.06	(0.00,0.16)
Subm.L.V10	-0.16	(−0.28,−0.05)	0.00	(−0.00,0.01)	0.02	(0.02,0.02)	0.01	(0.01,0.02)	0.01	(−0.10,0.08)	0.03	(−0.07,0.12)	0.01	(−0.03,0.06)	0.10	(0.00,0.23)
Subm.L.V30	0.20	(0.07,0.38)	0.00	(−0.00,0.01)	0.02	(0.01,0.02)	0.01	(0.01,0.03)	-0.04	(−0.10,0.03)	0.00	(−0.07,0.07)	0.01	(−0.05,0.06)	0.04	(0.00,0.30)
Subm.L.V50	-0.52	(−0.56,−0.48)	0.01	(−0.00,0.02)	0.05	(0.04,0.05)	0.03	(0.02,0.05)	0.09	(0.02,0.16)	0.08	(−0.02,0.15)	0.04	(−0.01,0.08)	0.00	(0.00,0.03)
Subm.R.Dm	-0.49	(−0.57,−0.41)	0.01	(0.00,0.02)	0.07	(0.06,0.07)	0.05	(0.03,0.07)	0.08	(0.04,0.11)	0.07	(0.03,0.11)	0.09	(0.05,0.15)	0.02	(0.00,0.11)
Subm.R.V10	-0.00	(−0.09,0.09)	0.00	(−0.00,0.01)	0.02	(0.01,0.02)	0.01	(0.00,0.01)	0.00	(−0.05,0.05)	0.03	(−0.03,0.10)	0.01	(−0.04,0.06)	0.00	(0.00,0.00)
Subm.R.V30	-0.21	(−0.32,−0.12)	0.00	(−0.00,0.01)	0.01	(0.00,0.01)	0.01	(−0.00,0.01)	-0.02	(−0.12,0.03)	0.01	(−0.08,0.05)	-0.02	(−0.07,0.03)	0.05	(0.00,0.12)
Subm.R.V50	0.33	(0.29,0.39)	0.01	(0.00,0.05)	0.06	(0.05,0.07)	0.05	(0.02,0.06)	0.12	(0.01,0.21)	0.10	(−0.01,0.19)	0.07	(0.03,0.11)	0.02	(0.00,0.08)
Parotid.L.Dm	-0.03	(−0.18,0.12)	0.01	(0.00,0.02)	0.04	(0.03,0.04)	0.03	(0.01,0.04)	0.01	(−0.02,0.05)	0.04	(−0.03,0.09)	0.04	(−0.01,0.08)	0.00	(0.00,0.01)
Parotid.L.V10	0.46	(0.40,0.52)	0.04	(0.01,0.08)	0.08	(0.07,0.08)	0.07	(0.05,0.09)	0.01	(−0.07,0.17)	0.02	(−0.05,0.11)	0.09	(0.05,0.14)	0.04	(0.00,0.08)
Parotid.L.V30	0.01	(−0.06,0.07)	0.01	(0.00,0.03)	0.01	(0.00,0.02)	0.01	(0.00,0.02)	-0.01	(−0.09,0.04)	0.02	(−0.05,0.09)	0.01	(−0.04,0.05)	0.00	(0.00,0.03)
Parotid.L.V50	-0.10	(−0.16,−0.05)	0.01	(0.00,0.03)	0.02	(0.01,0.03)	0.02	(0.01,0.03)	0.05	(−0.01,0.08)	0.06	(−0.01,0.10)	0.02	(−0.01,0.05)	0.01	(0.00,0.03)
Parotid.R.Dm	1.20	(1.02,1.44)	0.03	(0.01,0.08)	0.06	(0.06,0.07)	0.05	(0.03,0.07)	0.03	(0.01,0.06)	0.07	(0.01,0.13)	0.07	(0.03,0.11)	0.01	(0.00,0.06)
Parotid.R.V10	-0.36	(−0.45,−0.28)	0.01	(0.00,0.02)	0.03	(0.03,0.04)	0.02	(0.01,0.04)	-0.03	(−0.11,0.05)	0.01	(−0.10,0.09)	0.03	(−0.01,0.08)	0.00	(0.00,0.04)
Parotid.R.V30	-0.65	(−0.74,−0.55)	0.01	(0.00,0.03)	0.03	(0.02,0.04)	0.02	(0.01,0.04)	0.04	(−0.05,0.10)	0.06	(−0.01,0.12)	0.04	(−0.01,0.08)	0.00	(0.00,0.02)
Parotid.R.V50	-0.11	(−0.20,−0.03)	0.07	(0.04,0.09)	0.08	(0.08,0.09)	0.08	(0.06,0.09)	0.09	(0.05,0.17)	0.10	(0.05,0.14)	0.07	(0.04,0.11)	0.08	(0.05,0.10)
PCM.Sup.Dm	0.61	(0.52,0.71)	0.01	(0.00,0.03)	0.08	(0.08,0.09)	0.06	(0.03,0.08)	0.10	(0.04,0.14)	0.10	(0.05,0.15)	0.10	(0.05,0.14)	0.01	(0.00,0.08)
PCM.Sup.V10	-1.63	(−1.81,−1.45)	0.00	(−0.00,0.01)	-0.01	(−0.02,−0.01)	-0.00	(−0.02,0.01)	-0.03	(−0.17,0.02)	-0.02	(−0.16,0.05)	-0.06	(−0.15,0.01)	0.00	(0.00,0.00)
PCM.Sup.V30	-0.43	(−0.50,−0.35)	0.00	(0.00,0.01)	0.03	(0.03,0.04)	0.02	(0.01,0.03)	0.06	(−0.03,0.12)	0.06	(−0.01,0.09)	0.03	(−0.04,0.08)	0.00	(0.00,0.00)
PCM.Sup.V50	-0.24	(−0.29,−0.20)	0.04	(0.01,0.09)	0.11	(0.11,0.12)	0.10	(0.07,0.14)	0.19	(0.11,0.26)	0.17	(0.09,0.25)	0.11	(0.07,0.14)	0.04	(0.00,0.09)
PCM.Med.Dm	0.62	(0.52,0.71)	0.38	(0.31,0.43)	0.13	(0.12,0.14)	0.18	(0.12,0.28)	0.17	(0.08,0.47)	0.15	(0.08,0.33)	0.18	(0.12,0.24)	0.44	(0.37,0.50)
PCM.Med.V10	0.88	(0.73,1.03)	0.00	(−0.00,0.01)	0.05	(0.04,0.05)	0.03	(0.01,0.04)	0.21	(0.04,0.60)	0.14	(0.01,0.50)	0.12	(0.07,0.19)	0.89	(0.75,1.04)
PCM.Med.V30	1.43	(1.32,1.55)	0.01	(0.00,0.02)	0.06	(0.06,0.07)	0.05	(0.03,0.07)	0.11	(−0.01,0.61)	0.09	(−0.01,0.35)	0.13	(0.07,0.22)	0.08	(0.00,0.21)
PCM.Med.V50	-0.52	(−0.57,−0.48)	0.01	(0.00,0.02)	0.05	(0.03,0.05)	0.03	(0.01,0.05)	0.09	(−0.14,0.23)	0.10	(−0.07,0.24)	0.03	(−0.02,0.08)	0.00	(0.00,0.00)
PCM.Inf.Dm	2.33	(2.28,2.40)	0.28	(0.22,0.37)	0.14	(0.13,0.16)	0.18	(0.13,0.26)	0.13	(0.04,0.29)	0.15	(0.06,0.36)	0.16	(0.09,0.22)	0.37	(0.33,0.40)
PCM.Inf.V10	0.29	(0.17,0.40)	0.00	(−0.00,0.01)	0.02	(0.02,0.03)	0.01	(0.00,0.02)	0.48	(0.11,0.85)	0.28	(−0.08,0.78)	0.23	(0.15,0.31)	0.60	(0.49,0.72)
PCM.Inf.V30	-0.79	(−0.84,−0.75)	-0.03	(−0.08,−0.00)	-0.05	(−0.06,−0.04)	-0.04	(−0.06,−0.02)	-0.07	(−0.15,0.01)	-0.05	(−0.19,0.02)	-0.05	(−0.12,0.00)	0.00	(0.00,0.00)
PCM.Inf.V50	-1.15	(−1.21,−1.09)	0.01	(0.00,0.03)	0.07	(0.06,0.07)	0.05	(0.02,0.07)	0.15	(0.01,0.23)	0.11	(−0.06,0.22)	0.06	(0.02,0.10)	0.01	(0.00,0.05)
Supraglottic.Dm	0.41	(0.33,0.48)	0.07	(0.03,0.12)	0.12	(0.12,0.13)	0.12	(0.09,0.17)	0.12	(0.02,0.30)	0.12	(0.03,0.29)	0.14	(0.10,0.20)	0.05	(0.01,0.15)
Supraglottic.V10	-0.30	(−0.41,−0.21)	0.00	(−0.00,0.01)	0.03	(0.03,0.03)	0.01	(0.01,0.02)	0.35	(−0.15,0.63)	0.25	(−0.04,0.62)	0.10	(0.05,0.16)	0.00	(0.00,0.00)
Supraglottic.V30	-0.53	(−0.58,−0.47)	-0.01	(−0.05,0.00)	-0.04	(−0.05,−0.03)	-0.03	(−0.06,−0.00)	-0.04	(−0.25,0.07)	-0.01	(−0.14,0.07)	-0.05	(−0.11,0.01)	0.00	(0.00,0.00)
Supraglottic.V50	0.24	(0.18,0.28)	0.10	(0.06,0.13)	0.11	(0.10,0.11)	0.10	(0.08,0.12)	0.17	(0.08,0.24)	0.13	(0.00,0.22)	0.11	(0.06,0.15)	0.05	(0.01,0.09)
OralCavity.Ext.Dm	0.66	(0.55,0.78)	0.07	(0.01,0.18)	0.18	(0.17,0.19)	0.17	(0.12,0.22)	0.20	(0.15,0.25)	0.16	(0.09,0.27)	0.22	(0.17,0.29)	0.13	(0.00,0.29)
OralCavity.Ext.V10	0.56	(0.45,0.71)	0.00	(−0.00,0.01)	0.02	(0.02,0.03)	0.02	(0.00,0.02)	0.00	(−0.09,0.05)	0.03	(−0.06,0.08)	0.03	(−0.02,0.07)	0.01	(0.00,0.07)
OralCavity.Ext.V30	0.44	(0.37,0.49)	0.17	(0.11,0.24)	0.14	(0.13,0.15)	0.15	(0.11,0.20)	0.20	(0.14,0.34)	0.17	(0.08,0.27)	0.18	(0.14,0.23)	0.38	(0.28,0.46)
OralCavity.Ext.V50	0.59	(0.52,0.67)	0.58	(0.53,0.61)	0.27	(0.26,0.29)	0.34	(0.27,0.45)	0.31	(0.20,0.46)	0.25	(0.11,0.43)	0.23	(0.17,0.29)	0.64	(0.57,0.71)
GlotticArea.Dm	-0.83	(−0.90,−0.77)	0.01	(−0.00,0.01)	0.04	(0.04,0.05)	0.03	(0.01,0.04)	0.03	(−0.04,0.19)	0.02	(−0.08,0.18)	0.05	(0.01,0.10)	0.01	(0.00,0.07)
GlotticArea.V10	0.82	(0.67,1.04)	0.00	(−0.00,0.01)	0.02	(0.02,0.02)	0.01	(0.00,0.02)	0.48	(0.06,0.88)	0.28	(−0.10,0.77)	0.20	(0.13,0.27)	0.47	(0.36,0.58)
GlotticArea.V30	0.18	(0.14,0.22)	-0.02	(−0.06,−0.00)	-0.05	(−0.07,−0.04)	-0.05	(−0.07,−0.03)	-0.10	(−0.21,−0.03)	-0.09	(−0.18,−0.01)	-0.06	(−0.10,−0.01)	0.00	(0.00,0.00)
GlotticArea.V50	0.54	(0.50,0.58)	0.06	(0.02,0.09)	0.09	(0.08,0.10)	0.08	(0.06,0.11)	0.10	(−0.02,0.17)	0.09	(−0.01,0.18)	0.08	(0.04,0.13)	0.22	(0.19,0.25)
DYSFAGIE..BSL.2	0.16	(0.16,0.17)	0.14	(0.12,0.15)	0.15	(0.15,0.16)	0.15	(0.14,0.16)	0.21	(0.16,0.24)	0.17	(0.07,0.24)	0.11	(0.08,0.15)	0.19	(0.18,0.19)
DYSFAGIE..BSL.3	0.35	(0.34,0.36)	0.31	(0.30,0.32)	0.30	(0.29,0.31)	0.31	(0.29,0.32)	0.38	(0.32,0.41)	0.29	(0.14,0.41)	0.23	(0.19,0.27)	0.36	(0.35,0.36)
